# Tuberculosis in migrants moving from high-incidence to low-incidence countries: a population-based cohort study of 519 955 migrants screened before entry to England, Wales, and Northern Ireland

**DOI:** 10.1016/S0140-6736(16)31008-X

**Published:** 2016-11-19

**Authors:** Robert W Aldridge, Dominik Zenner, Peter J White, Elizabeth J Williamson, Morris C Muzyamba, Poonam Dhavan, Davide Mosca, H Lucy Thomas, Maeve K Lalor, Ibrahim Abubakar, Andrew C Hayward

**Affiliations:** a Centre for Public Health Data Science, Institute of Health Informatics, University College London, London, UK; b Farr Institute of Health Informatics Research, University College London, London, UK; c Institute for Global Health, University College London, London, UK; d Centre for Infectious Disease Surveillance and Control, Public Health England, London, UK; e MRC Centre for Outbreak Analysis and Modelling and NIHR Health Protection Research Unit in Modelling Methodology, Imperial College London, London, UK; f Migration Health Division, International Organization for Migration, Geneva, Switzerland

## Abstract

**Background:**

Tuberculosis elimination in countries with a low incidence of the disease necessitates multiple interventions, including innovations in migrant screening. We examined a cohort of migrants screened for tuberculosis before entry to England, Wales, and Northern Ireland and tracked the development of disease in this group after arrival.

**Methods:**

As part of a pilot pre-entry screening programme for tuberculosis in 15 countries with a high incidence of the disease, the International Organization for Migration screened all applicants for UK visas aged 11 years or older who intended to stay for more than 6 months. Applicants underwent a chest radiograph, and any with results suggestive of tuberculosis underwent sputum testing and culture testing (when available). We tracked the development of tuberculosis in those who tested negative for the disease and subsequently migrated to England, Wales, and Northern Ireland with the Enhanced Tuberculosis Surveillance system. Primary outcomes were cases of all forms of tuberculosis (including clinically diagnosed cases), and bacteriologically confirmed pulmonary tuberculosis.

**Findings:**

Our study cohort was 519 955 migrants who were screened for tuberculosis before entry to the UK between Jan 1, 2006, and Dec 31, 2012. Cases notified on the Enhanced Tuberculosis Surveillance system between Jan 1, 2006, and Dec 31, 2013, were included. 1873 incident cases of all forms of tuberculosis were identified, and, on the basis of data for England, Wales, and Northern Ireland, the estimated incidence of all forms of tuberculosis in migrants screened before entry was 147 per 100 000 person-years (95% CI 140–154). The estimated incidence of bacteriologically confirmed pulmonary tuberculosis in migrants screened before entry was 49 per 100 000 person-years (95% CI 45–53). Migrants whose chest radiographs were compatible with active tuberculosis but with negative pre-entry microbiological results were at increased risk of tuberculosis compared with those with no radiographic abnormalities (incidence rate ratio 3·2, 95% CI 2·8–3·7; p<0·0001). Incidence of tuberculosis after migration increased significantly with increasing WHO-estimated prevalence of tuberculosis in migrants' countries of origin. 35 of 318 983 pre-entry screened migrants included in a secondary analysis with typing data were assumed index cases. Estimates of the rate of assumed reactivation tuberculosis ranged from 46 (95% CI 42–52) to 91 (82–102) per 100 000 population.

**Interpretation:**

Migrants from countries with a high incidence of tuberculosis screened before being granted entry to low-incidence countries pose a negligible risk of onward transmission but are at increased risk of tuberculosis, which could potentially be prevented through identification and treatment of latent infection in close collaboration with a pre-entry screening programme.

**Funding:**

Wellcome Trust, UK National Institute for Health Research, UK Medical Research Council, Public Health England, and Department of Health Policy Research Programme.

## Introduction

Several countries have achieved low tuberculosis incidence while having large populations of migrants from countries with high tuberculosis burdens.^1^, ^2^, ^3^ In many of these countries with low burdens of tuberculosis, a large proportion of disease now occurs in individuals born outside the country.^4^, ^5^ Elimination of tuberculosis in countries with low incidence of the disease is expected to require multiple interventions, including, but not limited to, innovation in screening of migrants.

A systematic review^6^ published in 2014 showed that, in pre-entry screening of migrants before travel to low-incidence countries, the largest number of cases was detected when screening was done in high-incidence countries. One-off pre-entry screening for active tuberculosis in migrants will detect only disease prevalent at the time of screening. Few linked data are available for incident tuberculosis after migration to low-incidence countries in populations screened before entry, and even fewer data are available for risk factors for subsequent development of disease in this screened population. Investigators in previous studies focused on prevalent cases detected during pre-entry screening,^6^ used national tuberculosis notification data alone with no linkage to pre-entry screening records,^7^ or followed up a selected cohort of individuals from a few countries or subnationally after arrival.^8^, ^9^, ^10^ Thus, the most effective approach to reduction of the disease burden in migrants from high-incidence to low-incidence countries—including latent tuberculosis screening and treatment, active case finding, and improvement of health-care access—is uncertain.

**Research in context**

**Evidence before this study**

We updated our recent systematic review of pre-entry screening for tuberculosis to include new articles published until Nov 19, 2015. In our original search, we searched for reports published after Jan 1, 1980, in MEDLINE, Embase, LILACS, Cochrane Infectious Diseases Group Specialized Register, Cochrane Library, Conference Proceedings Citation Index—Science, and Conference Proceedings Citation Index—Social Science & Humanities. Search terms covered the populations of interest (migrants, refugees, asylum seekers, new entrants, undocumented migrants), the intervention (pre-entry screening), and standard terms for tuberculosis. Reference lists of included studies were hand searched to identify further relevant work. In the updated search, we used our original search terms, including “migrants”, “pre-entry screening”, and standard terms for tuberculosis. The updated search was done in MEDLINE and Embase. The prevalence of culture-confirmed cases of tuberculosis detected before entry to countries with a low burden of tuberculosis increased with increased prevalence in the country of origin, but we found little evidence of systematic follow-up of migrants after arrival at a national level, and no comprehensive studies that included molecular epidemiological data to examine reactivation and transmission after arrival.

**Added value of this study**

In our study we systematically estimate the incidence of, and risk factors for, all forms of tuberculosis and bacteriologically confirmed pulmonary tuberculosis (ie, those cases that could transmit infection) in migrants screened before entry and in whom disease was diagnosed after arrival in a country with a low incidence of tuberculosis. Risk of active tuberculosis (all forms) diagnosed after arrival increased with incidence in the country of origin and was higher in migrants with chest radiographs compatible with active tuberculosis, but in whom disease was not necessarily bacteriologically confirmed. Our analysis included comprehensive molecular typing data for culture-confirmed cases since 2010, and showed that migrants with chest radiographs compatible with active tuberculosis, but not diagnosed with tuberculosis before entry, were at increased risk of disease assumed to be a result of reactivation. The incidence of assumed index cases was low, suggesting that migrants screened before entry to low-incidence countries pose a negligible public health risk in terms of transmission. The introduction of culture testing on sputum samples to the pre-entry protocol was associated with a decreased risk of tuberculosis notification after migration, supporting changes by the UK and US programmes.

**Implications of all the available evidence**

Migrants screened for tuberculosis before entry to the UK have a continuing risk of developing disease, much of which could plausibly be prevented through identification and treatment of latent infection. Given that incidence peaks in the fourth year after arrival, migrants from countries with a high incidence of tuberculosis could be included in a catch-up screening programme for latent tuberculosis. Improved testing and treatment of latent infection could mitigate some of the health impact of tuberculosis in migrant groups.

**Figure 1:**
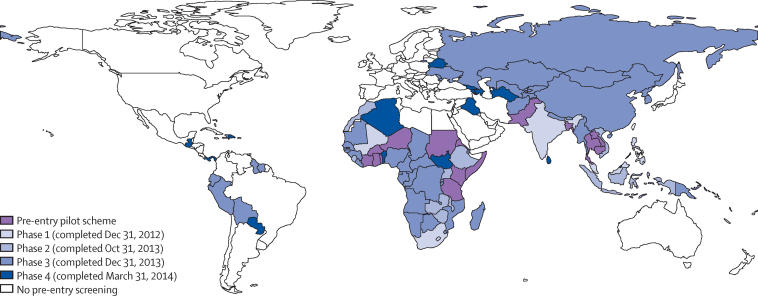
Locations of pre-entry screening sites Includes both International Organization for Migration sites and other sites. The pre-entry pilot scheme was done in Bangladesh, Burkina Faso, Cambodia, Côte d'Ivoire, Eritrea, Ghana, Kenya, Laos, Niger, Pakistan, Somalia, Sudan, Tanzania, Thailand, and Togo. Pre-entry screening was subsequently rolled out to 101 countries in four phases between May, 2012, and March 31, 2014.

A UK pilot programme of pre-entry screening was done in 15 countries from 2005, and subsequently rolled out to 101 countries between May, 2012, and March 31, 2014 (figure 1).^11^ We use data from this pilot programme to create the largest cohort so far of migrants screened for active tuberculosis before entering a country with low incidence of tuberculosis, generate national estimates of incidence after migration, and identify risk factors for subsequent development of disease. We used strain typing data to identify tuberculosis cases that were probably due to reactivation of imported latent infection rather than acquisition in England, Wales, or Northern Ireland. We also identified index cases for clusters of disease, and ascertained the characteristics of such cases.

## Methods

### Study design and participants

We did a population-based cohort study of migrants to the UK screened before entry for active tuberculosis. Screening occurred between Jan 1, 2006, and Dec 31, 2012, and was done by the International Organization for Migration (IOM) as part of a pilot pre-entry screening programme in 15 countries with a high incidence of tuberculosis—Bangladesh, Burkina Faso, Cambodia, Côte d'Ivoire, Eritrea, Ghana, Kenya, Laos, Niger, Pakistan, Somalia, Sudan, Tanzania, Thailand, and Togo (figure 1). All visa applicants from these countries who were 11 years or older and intended to stay for more than 6 months were included in this study.

All participants were screened for active tuberculosis at specified health centres (when such health centres were unavailable in the country of origin, participants had to travel to other countries for screening).^12^ Briefly, they underwent a standard chest radiograph, and any with radiological findings compatible with tuberculosis (appendix) were required to undergo sputum testing for tuberculosis. All testing was done by IOM staff. Culture testing for those with radiological evidence of disease was phased in gradually across sites from 2007, but became a requirement at all sites from 2013. Applicants without radiological changes compatible with tuberculosis, and those with radiological change suggesting tuberculosis but negative sputum smears and cultures, were provided with medical clearance certificates. Those diagnosed with active tuberculosis in their country of origin were denied a medical clearance certificate (necessary to obtain a visa) and required to complete treatment before repeat screening. We have previously reported findings about participants diagnosed in their country of origin.^11^ In this Article, we focus on participants who tested negative for tuberculosis at pre-entry screening, but were subsequently diagnosed with the disease after arrival in England, Wales, or Northern Ireland. Data for all migrants screened before entry during the study period were used to link records to the Enhanced Tuberculosis Surveillance (ETS) system^13^ to identify subsequent cases of tuberculosis notified in England, Wales, or Northern Ireland. The ETS contains data for all tuberculosis cases notified in England, Wales, and Northern Ireland, including both microbiologically confirmed cases and clinically diagnosed cases being treated with a full course of tuberculosis drugs.

Ethical approval was received for this analysis from the University College London research ethics committee (3294/002). Data were stored and analysed at Public Health England, which has authority under the UK Health and Social Care Act 2012 to hold and analyse national surveillance data (including tuberculosis pre-entry screening programme data) for public health and research purposes. As part of the screening process, migrants consented for their data to be used by Public Health England and NHS England.

### Outcomes

Our primary outcomes were cases of all forms of tuberculosis (including clinically diagnosed cases), and bacteriologically confirmed pulmonary tuberculosis. Definitions and cases for the primary outcomes were taken from the ETS.

Secondary outcomes were cases of tuberculosis assumed to be due to reactivation on the basis of molecular typing data (subsequently referred to as assumed reactivation cases or reactivation cases), and cases assumed to have led to transmission in England, Wales, and Northern Ireland because they were the first tuberculosis case in a molecular strain typing cluster (subsequently referred to as assumed index cases or index cases). Reactivation cases represent disease that is potentially preventable through treatment of latent infection in migrants, and index cases lead to transmission that is potentially preventable. Individuals classified as index cases and reactivation cases were culture confirmed and had mycobacterial interspersed repetitive units and variable number tandem repeat (MIRU–VNTR) profiles, with at least 23 complete loci notified in the ETS between Jan 1, 2010 (when strain-typing data began to be systematically collected), and Dec 31, 2013.^14^ The index cases were identified by recorded date of notification and had a unique 23 MIRU–VNTR strain type compared with all previously notified cases, but shared their strain type with one or more subsequently notified cases, suggesting possible transmission. Reactivation cases were defined as all cases with a unique 23 MIRU–VNTR strain type. No country of birth or geographical restrictions were placed on ETS data (ie, people from the UK and migrants from countries other than the 15 pilot sites were also included). All migrants screened by IOM between Jan 1, 2009, and Dec 31, 2012, were included in the analysis of secondary outcomes. Patients screened before 2009 were excluded to reduce potential biases.

### Statistical analysis

A full description of the data sources of variables, methods of assessment, and details of subgroups chosen for the analysis are presented in the appendix. We used a validated probabilistic linkage software program,^15^ Enhanced Matching System, to identify active tuberculosis cases notified in England, Wales, and Northern Ireland in the migrant cohort by linking the IOM database of migrants screened before entry between Jan 1, 2006, and Dec 31, 2012, to ETS-notified cases between Jan 1, 2006, and Dec 31, 2013. Cases notified in England, Wales, and Northern Ireland within 90 days of the issue of a medical certificate of clearance were assumed to be prevalent (not incident) tuberculosis missed by pre-entry screening (ie, missed prevalent cases), and were excluded from incidence rate analyses. Duplicate records were also removed (appendix).

Individuals in our cohort were at risk of tuberculosis notification from the time they were given a certificate of clearance until the first of tuberculosis notification, death, emigration, or the end of the follow-up period (ie, Dec 31, 2013). Because data were unavailable to indicate immigration to Scotland (although a full UK dataset was used, migrants living in Scotland who were diagnosed with tuberculosis were not detected by our probabilistic linkage software because we did not have their personal identifiable variables), these events were accounted for probabilistically by multiple imputation. Data for long-term international migration suggested that 7·3% of migrants entering the UK between 2006 and 2012 would reside in Scotland.^16^ Therefore, each imputation model was programmed to randomly select 92·7% of migrants issued with a medical certificate of clearance to enter the cohort as a resident in England, Wales, or Northern Ireland.

We also used imputation models to account for dates of death and emigration out of the UK to create ten imputed datasets, each of which we analysed separately. Mortality data for England and Wales from 2009 were used in the models for dates of death, and data for UK entry clearance visas, which detailed length of stay by type of visa and year of issue, were used in the models for emigration from the UK (appendix). Analyses of the ten imputed datasets were combined by Rubin's rules^17^ to account for uncertainty in the imputed information (appendix).

Each imputed dataset was analysed as follows. We used univariable and multivariable Poisson regression models to identify risk factors for the primary and secondary outcomes. Multivariable results were adjusted for clustering by individual to take into account repeated entries by migrants into the cohort (as a result of migrants entering, leaving, and entering again with a new visa). In a further analysis, data were stratified by years since entry to examine incidence over time. We present our results as incidence per 100 000 person-years and incidence rate ratios (IRRs), with 95% CIs and two-sided p values.

We did several sensitivity analyses to examine the rules used in imputation, deduplication, the period used for the definition of prevalent cases, and that used for strain typing data. To create a crude upper bound of reactivation incidence, we assumed that the proportion of cases with a unique strain type in untyped cases was similar to that in typed cases. These sensitivity analyses and the results are described in full in the appendix. We used Stata (version 13.1) for all statistical analyses.

### Role of the funding source

The funders of this study had no role in study design; data collection, analysis, or interpretation; or writing of the report. The corresponding author had full access to all the data in the study and the final responsibility to submit for publication.

## Results

**Figure 2:**
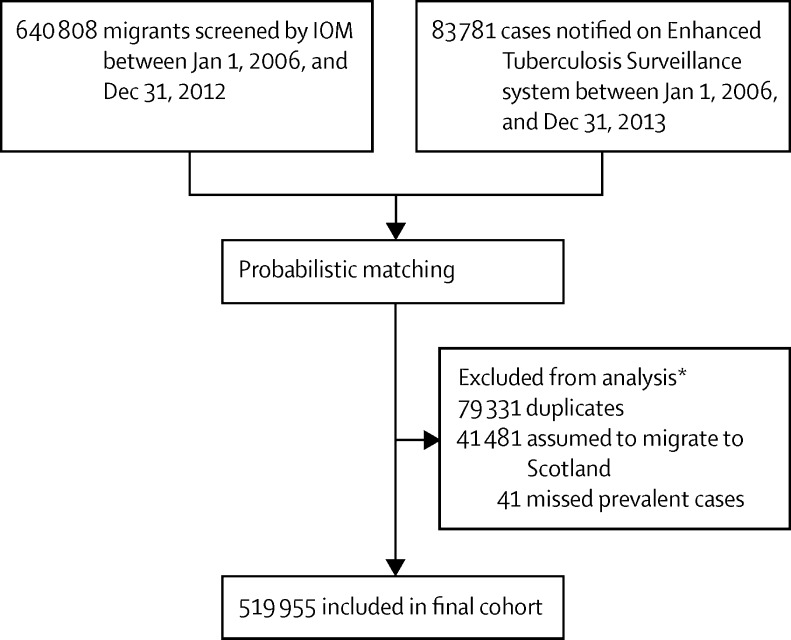
Study participant flow chart IOM=International Organization for Migration. *Numbers assumed to migrate to Scotland varied by imputation, and the sum of those excluded does not equal the difference between total visa applicants and the number of migrants included in the final cohort, because groups were not mutually exclusive.

Between Jan 1, 2006, and Dec 31, 2012, the IOM screened 640 808 visa applicants before entry (figure 2), whose records were probabilistically matched to the ETS to identify tuberculosis cases with dates of illness onset between Jan 1, 2006, and Dec 31, 2013. After duplicates (79 331) and missed prevalent cases (41) were excluded, 561 436 migrants were left. Assuming that 7·3% of migrants moved to Scotland, 519 955 migrant entries to England, Wales, and Northern Ireland comprised the study cohort, representing 514 968 individual migrants.

The total length of follow-up for the cohort was 1 275 569 person-years, with a mean follow-up of 2·45 years per person. Of the 41 missed prevalent cases identified in the 90 days after migration, 15 (37%) had pre-entry chest radiographs compatible with active tuberculosis (but had bacteriologically negative results before entry). 1873 incident cases of all forms of tuberculosis were identified (ie, diagnosed after migration), a crude incidence rate of 147 per 100 000 person-years (95% CI 140–154). The estimated incidence of bacteriologically confirmed pulmonary tuberculosis in migrants screened before entry was 49 per 100 000 person-years (95% CI 45–53).

In a multivariable risk factor analysis adjusted for age and sex (table 1), self-report of close or household contact with a case of tuberculosis (IRR 3·0, 95% CI 1·8–5·1; p<0·0001) and having a screening chest radiograph compatible with active tuberculosis but negative microbiology results at the time of screening (3·2, 2·8–3·7; p<0·0001) were strongly associated with increased risk of all forms of tuberculosis notified in England, Wales, and Northern Ireland. Migrants from countries with WHO-estimated tuberculosis prevalences of 40–149 per 100 000 people (0·3, 0·2–0·4; p<0·0001) and those from countries with prevalences of 150–349 per 100 000 people (0·6, 0·5–0·6; p<0·0001) were at lower risk of developing tuberculosis than were migrants from countries with prevalences greater than 350 per 100 000. Visa category and being screened at a site where culture testing of sputum samples was done were also significantly associated with tuberculosis risk (table 1).

**Figure 3:**
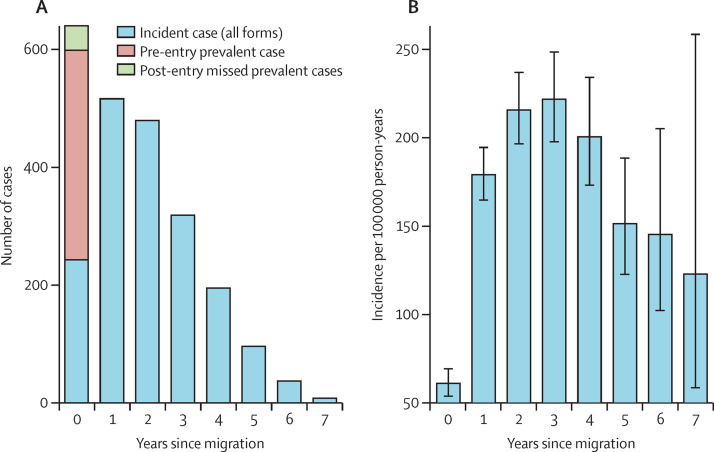
Cases of tuberculosis notified in migrants to England, Wales, and Northern Ireland (A), and incidence rates for tuberculosis (B), by time since entry (A) Includes 439 pre-entry prevalent cases detected between Jan 1, 2006, and Dec 31, 2012, post-entry missed prevalent cases (41 cases notified within 90 days after migration), and all tuberculosis cases (1873 cases) notified in the UK among migrants by year since migration. The error bars in (B) are 95% CIs.

When all 2353 cases of tuberculosis detected both before entry and after migration were considered, most (1873 [79·6%]) were incident cases notified in England, Wales, and Northern Ireland, with fewer pre-entry prevalent cases (439 [18·7%]) and some missed prevalent cases noted with 90 days of migration (41 [1·7%]; figure 3A). The total number of tuberculosis cases in migrants declined each year since migration when prevalent cases detected at pre-entry screening (who were excluded from the cohort because they were declined medical certificates of clearance) and missed prevalent cases detected after entry were included (figure 3). Accounting for person time at risk within the cohort (and excluding pre-entry and post-entry prevalent cases) the incidence of all forms of tuberculosis was lowest in the first 12 months after migration (61 per 100 000 person-years, 95% CI 54–69), peaked in the fourth year (222, 198–249), and then gradually fell (figure 3B).

318 983 migrants screened before entry with 648 385 person-years' follow-up within the cohort formed the basis of our examination of assumed reactivation and index cases. 650 (68·3%) of 952 notified cases of disease in this cohort were culture confirmed, and strain typing results were available for 529 (81·4%) of the 650. 301 cases of assumed reactivation were noted, with crude estimated incidence of 46 per 100 000 person-years (95% CI 42–52). A crude upper bound of reactivation, assuming that the proportion of cases with a unique strain type in untyped cases was similar to that in typed cases, provided an estimate of 91 per 100 000 person-years (95% CI 82–102). After adjustment for age and sex (table 2), compared with migrants from countries with tuberculosis prevalences greater than 350 per 100 000 people, those from countries with a prevalence of 40–149 per 100 000 were at lower risk of reactivation (IRR 0·2, 95% CI 0·1–0·9; p=0·034), as were those from countries with a prevalence of 150–349 per 100 000 (0·3, 0·2–0·6; p<0·0001; table 2). Migrants with a chest radiograph classified as compatible with tuberculosis (but that was not bacteriologically confirmed at the time of screening) were at increased risk of reactivation (3·9, 2·7–5·5; p<0·0001; table 2).

35 migrants were assumed index cases for onward transmission, resulting in an estimated crude rate of five per 100 000 person-years (95% CI 4–8). The crude incidence rate for index cases was highest in those with chest radiographs suggestive of active tuberculosis but with negative bacteriology at the time of screening (36, 95% CI 19–68); this group had increased risk (IRR 9·6, 95% CI 4·4–20·7; p<0·0001) compared with those with no abnormalities, after adjustment for age and sex.

The results of our analyses were stable across a range of sensitivity analyses done to test the data and key assumptions in the imputation—full results are presented and discussed in the appendix.

## Discussion

We present data from an analysis of a cohort of more than half a million migrants screened for tuberculosis from 15 high-incidence countries between Jan 1, 2006, and Dec 31, 2012. Our data suggest that migrants screened before entry pose a negligible risk in terms of onwards transmission within the host country, but their individual risk remains increased, which presents risk to their own health.

Our results show that individuals with chest radiographs classified as compatible with active tuberculosis (but not diagnosed as tuberculosis before entry) and those reporting a history of close or household contact with a case of tuberculosis before migration were associated with increased risk of tuberculosis (all forms) and bacteriologically confirmed pulmonary disease. Migrants who were screened at locations where sputum samples were culture tested had a lower risk of developing all forms of tuberculosis after arrival in England, Wales, and Northern Ireland than those who were not. Incidence of all forms of tuberculosis was lowest in the first year after arrival and peaked in the fourth year, before gradually declining, although 95% CIs for estimates in later years were wide because of decreasing cohort size. Rates of assumed index cases were low, with only 35 cases identified in total.

All visa applicants from the 15 countries taking part in the pre-entry screening pilot were included in our analysis, and therefore our results are highly representative of migrants from these locations applying to stay for 6 months or longer. We identified migrants screened before entry, not only those self-reporting birth outside the UK, and as a result we provide the first national estimates of, and risk factors for, incident tuberculosis in migrants screened before entry.

We also provide the first national description of strain typing data in migrants screened for active tuberculosis in the pre-entry programme, based on MIRU–VNTR. Compared with restriction fragment length polymorphism, MIRU–VNTR analysis does not require large quantities of DNA (it is a PCR-based method), is quicker, and provides a result that can be digitised and exchanged between national tuberculosis control programmes more easily. Spoligotyping, another PCR-based method, has less discriminatory power than restriction fragment length polymorphism, whereas MIRU–VNTR analysis has equivalent or improved power compared with analysis of restriction fragment length polymorphisms. Additionally, MIRU–VNTR analysis is now used by most national tuberculosis control programmes that routinely do molecular testing of cases.^18^

Our study has some limitations. The data are highly representative of migrants to the UK intending to stay for more than 6 months, but do not include those intending shorter stays or undocumented migrants and asylum seekers.^19^ However, although undocumented migrants and asylum seekers are a particularly vulnerable group, their comparatively small numbers mean they account for low numbers of notified cases. Several risk factors for tuberculosis were included in this analysis, but no data were available for socioeconomic status, relevant clinical conditions (eg, HIV), lifestyle and behavioural risk factors (eg, smoking, problem drug and alcohol use), or history of imprisonment, all of which are associated with an increased risk of tuberculosis in the UK.^20^, ^21^, ^22^, ^23^, ^24^, ^25^, ^26^

Although there is a high level of certainty that individuals who receive an entry visa for the UK migrate,^27^, ^28^, ^29^ when and whether these individuals leave after their visa expires is less sure. We account for the uncertain duration of stay and death rates through imputation based on data for visa length and national death rates. Several sensitivity analyses of the impact of these assumptions were stable, which provides reassurance that these data and results are robust.

The strain typing data used in this analysis have several important limitations. Firstly, only 68·3% of notified cases were culture confirmed, 81·4% of which had strain typing results in the UK. Cases without a strain type were not classified with this approach. Furthermore, many of the cases that were not culture confirmed are also probably due to reactivation. As a result, there is under-ascertainment of assumed reactivation cases, making the estimates presented here lower bounds of true rates, although our estimated upper bound provides a likely ceiling to this estimate.^25^ Secondly, assumed index cases might have been underestimated because a unique strain might be involved in a cluster with another case that has not yet been reported. The sensitivity analysis examining clustering by time provides us with some confidence that, within this dataset of 4 years of data, this issue should be minimal.

Finally, whole-genome sequencing has shown that MIRU–VNTR strain typing does not provide a very high level of resolution, and that cases with identical strain types as identified by this process might not be part of the same transmission chain,^30^ which would lead to an overestimate in the number of index cases and further underestimation of the number of reactivation cases. Again, such a scenario is likely to have only a small impact on index case rates.^30^, ^31^, ^32^, ^33^ Overall, we believe that, as a result of these limitations, the incidence estimates for reactivation presented here are probably lower than the true values, and the magnitude of bias in assumed index cases is expected to be small.

Among people screened before entry, several groups are at higher risk of tuberculosis after migration, including those on family reunion visas, those from especially high-incidence countries, those with positive chest radiograph findings, and those reporting close or household contact with a case of tuberculosis before migration (table 1). For these groups, efforts should be made to facilitate access to health services, including access to latent tuberculosis screening, to reduce the risks of poorer health outcomes and transmission to others. Improved integration of pre-entry screening programmes and health services in the destination country will benefit migrants and local populations and improve targeted post-entry latent tuberculosis screening efforts. A cost-effectiveness analysis of these data and those from previous studies should also be done for active pre-entry screening to help to refine targeting screening programmes further.^6^, ^11^

Our results support continuation of pre-entry radiographic screening and culture-based investigation of people whose radiographs are consistent with tuberculosis. Screening for latent infection could be targeted at those from high-incidence countries and those with chest radiographs compatible with active tuberculosis but negative bacteriological results on initial screening. Screening for latent tuberculosis infection could be done alongside pre-entry screening or after migration. Uptake of such screening is likely to be higher if done alongside pre-entry screening, but uptake of treatment might be higher if screening is done after migration, when migrants are linked into health services. Given that incidence peaks 4 years after arrival, people who have migrated in the past 5 years from countries with very high incidences of tuberculosis might be targeted in a catch-up programme. Screening programmes for latent tuberculosis infection should be assessed and their cost-effectiveness should be determined; delivery models should then be adapted in light of the findings. The data-linkage approach we have developed could be used to support such assessments and to investigate broader health issues in migrant populations.

The fact that only 35 of more than 300 000 migrants screened before entry to England, Wales, and Northern Ireland were identified as assumed index cases suggests that, after screening, migrants pose a negligible public health risk in terms of transmission. Our study provides the basis for several evidence-based improvements to pre-entry screening of migrants, which, if implemented, will contribute to the target of tuberculosis elimination in low-incidence countries and improving the health of migrants.

## Figures and Tables

**Table 1:** Baseline characteristics, univariable, and multivariable analysis of incidence of tuberculosis and bacteriologically confirmed pulmonary tuberculosis in migrants screened before entry and notified in the Enhanced Tuberculosis Surveillance system after arrival

	**Migrants contributing (n=519 955) (%)**	**All notified cases of tuberculosis**				**Bacteriologically confirmed pulmonary tuberculosis**			
		Rate per 100 000 person-years (95% CI)	Univariable IRR (95% CI)	Multivariable IRR (95%CI)	p	Rate per 100 000 person-years (95% CI)	Univariable IRR (95% CI)	Multivariable IRR (95%CI)	p
**Age (years)**									
0–15	15 468 (3·0%)	101 (77–132)	0·7 (0·5–0·9)	0·8 (0·6–1·0)	0·057	37 (24–58)	0·7 (0·5–1·2)	0·9 (0·6–1·4)	0·677
16–44	490 806 (94·4%)	149 (142–156)	1·0	1·0	..	50 (46–54)	1·0	1·0	..
45–64	11 243 (2·2%)	146 (111–193)	1·0 (0·7–1·3)	1·0 (0·8–1·3)	0·941	35 (20–62)	0·7 (0·4–1·2)	0·7 (0·4–1·3)	0·271
>65	2438 (0·5%)	210 (127–347)	1·5 (0·9–2·5)	1·1 (0·7–2·0)	0·615	92 (43–194)	2·1 (1·0–4·6)	1·7 (0·7–3·8)	0·232
**Sex**									
Female	173 116 (33·3%)	133 (123–143)	1·0	1·0	..	45 (39–51)	1·0	1·0	..
Male	346 839 (66·7%)	157 (149–167)	1·2 (1·1–1·3)	1·0 (0·9–1·1)	0·533	52 (47–58)	1·2 (1·0–1·4)	0·9 (0·8–1·1)	0·357
**Close or household contact with a case of tuberculosis**									
No	518 735 (99·8%)	146 (140–153)	1·0	..	..	49 (45–53)	1·0	1·0	..
Yes	1220 (0·2%)	463 (279–770)	3·1 (1·9–5·2)	3·0 (1·8–5·1)	<0·0001	278 (144–535)	5·7 (2·9–11·0)	5·0 (2·6–9·7)	<0·0001
**Type of visa**									
Students	307 127 (59·1%)	161 (151–172)	1·0	1·0	..	58 (52–64)	1·0	1·0	..
Settlement and dependents	159 986 (30·8%)	128 (119–137)	0·8 (0·7–0·9)	0·8 (0·7–0·9)	0·0001	41 (37–47)	0·7 (0·6–0·9)	0·7 (0·6–0·8)	0·0001
Work	21 140 (4·1%)	174 (139–218)	1·1 (0·9–1·4)	1·0 (0·8–1·3)	0·722	53 (35–79)	0·9 (0·6–1·4)	0·9 (0·6–1·3)	0·559
Working holiday maker	17 526 (3·4%)	146 (94–226)	0·9 (0·6–1·4)	1·1 (0·7–1·7)	0·675	15 (4–58)	0·3 (0·1–1·0)	0·3 (0·1–1·2)	0·083
Family reunion	3989 (0·8%)	320 (244–419)	2·0 (1·5–2·6)	2·6 (1·9–3·5)	<0·0001	72 (41–128)	1·3 (0·7–2·2)	1·4 (0·8–2·6)	0·227
Other	10 187 (2·0%)	124 (81–191)	0·8 (0·5–1·2)	1·2 (0·8–1·9)	0·373	41 (20–87)	0·7 (0·3–1·5)	1·0 (0·5–2·2)	0·941
**Chest radiograph classification**									
No abnormality	489 733 (94·2%)	135 (129–142)	1·0	1·0	..	43 (40–47)	1·0	1·0	..
Compatible with tuberculosis	21 862 (4·2%)	426 (375–484)	3·2 (2·8–3·6)	3·2 (2·8–3·7)	<0·0001	177 (146–216)	4·1 (3·3–5·1)	4·2 (3·4–5·3)	<0·0001
Abnormality not tuberculosis	8360 (1·6%)	82 (52–130)	0·6 (0·4–1·0)	0·8 (0·5–1·3)	0·455	27 (12–61)	0·6 (0·3–1·4)	0·8 (0·4–1·9)	0·624
**WHO-estimated prevalence (per 100 000 people)**									
40–149	29 143 (5·6%)	40 (27–60)	0·2 (0·2–0·4)	0·3 (0·2–0·4)	<0·0001	17 (9–31)	0·3 (0·2–0·6)	0·4 (0·2–0·7)	0·002
150–349	75 294 (14·5%)	107 (94–122)	0·7 (0·6–0·8)	0·6 (0·5–0·6)	<0·0001	40 (32–49)	0·8 (0·6–1·0)	0·6 (0·5–0·8)	0·0005
>350	415 518 (79·9%)	162 (154–170)	1·0	1·0	..	53 (49–58)	1·0	1·0	..
**Sputum culture testing**									
No	179 935 (34·6%)	152 (143–163)	1·0	1·0	..	50 (44–56)	1·0	1·0	..
Yes	340 020 (65·4%)	143 (134–152)	0·9 (0·8–1·0)	0·9 (0·8–1·0)	0·003	49 (44–54)	1·0 (0·8–1·2)	0·9 (0·8–1·0)	0·152

Data for screening before entry are for Jan 1, 2006, to Dec 31, 2012; those for the Enhanced Tuberculosis Surveillance system are for Jan 1, 2006, to Dec 31, 2013. The overall rate among all notified cases of tuberculosis was 147 (95% CI 140–154) per 100 000 person-years; among cases of bacteriologically confirmed tuberculosis it was 49 (45–53) per 100 000 person-years. Data for incidence of tuberculosis include all forms of disease, including clinically diagnosed cases. Rows in which the IRRs=1·0 are the reference data. IRR=incidence rate ratio.

**Table 2:** Baseline characteristics, univariable, and multivariable analysis of incidence rates for assumed reactivation and assumed index cases of tuberculosis in migrants screened before entry and notified in the Enhanced Tuberculosis Surveillance system after arrival

	**Migrants contributing (n=318 983) (%)**	**Assumed reactivation**				**Assumed index cases**			
		Rate per 100 000 person-years (95% CI)	Univariable IRR (95% CI)	Multivariable IRR (95%CI)	p	Rate per 100 000 person years (95% CI)	Univariable IRR (95% CI)	Multivariable IRR (95% CI)	p
**Age (years)**									
0–15	9542 (3·0%)	12 (4–38)	0·3 (0·1–0·8)	0·4 (0·1–1·1)	0·073	4 (1–29)	0·7 (0·1–5·4)	1·2 (0·2–8·5)	0·845
16–44	301 358 (94·5%)	48 (43–54)	1·0	1·0	..	6 (4–8)	1·0	1·0	
45–64	6466 (2·0%)	33 (14–79)	0·7 (0·3–1·6)	0·9 (0·4–2·3)	0·868	..	..	..	..
>65	1617 (0·5%)	26 (4–185)	0·5 (0·1–3·9)	0·5 (0·1–3·7)	0·498	..	..	..	..
**Sex**									
Female	101 715 (31·9%)	33 (27–41)	1·0	1·0	..	3 (1–6)	1·0	1·0	..
Male	217 268 (68·1%)	54 (47–62)	1·6 (1·3–2·1)	1·1 (0·8–1·5)	0·47	7 (5–10)	2·8 (1·1–6·7)	1·9 (0·8–4·6)	0·14
**Close or household contact with a case of tuberculosis**									
No	318 126 (99·7%)	46 (41–52)	1·0	1·0		5 (4–8)	..	..	..
Yes	857 (0·3%)	55 (8–394)	1·2 (0·2–8·5)	1·3 (0·2–9·2)	0·803	..	..	..	..
**Type of visa**									
Students	206 142 (64·6%)	58 (50–66)	1·0	1·0		7 (5–10)	1·0	1·0	..
Settlement and dependents	94 118 (29·5%)	32 (26–40)	0·6 (0·4–0·7)	0·7 (0·5–0·9)	0·012	4 (2–7)	0·5 (0·2–1·1)	0·8 (0·4–1·8)	0·581
Work	10 578 (3·3%)	31 (14–69)	0·5 (0·2–1·2)	0·6 (0·3–1·2)	0·147	5 (1–37)	0·8 (0·1–5·6)	0·9 (0·1–5·6)	0·871
Working holiday maker	861 (0·3%)	..	..	..		..	..	..	..
Family reunion	2335 (0·7%)	73 (31–176)	1·3 (0·5–3·1)	3·1 (1·3–7·6)	0·013	..	..	..	..
Other	4949 (1·6%)	..	..	..	..	..	..	..	..
**Chest radiograph classification**									
No abnormality	302 364 (94·8%)	43 (38–48)	1·0	1·0	..	4 (3–6)	1·0	1·0	..
Compatible with tuberculosis	12 304 (3·9%)	154 (113–211)	3·6 (2·6–5·1)	3·9 (2·7–5·5)	<0·0001	36 (19–68)	8·4 (3·9–17·9)	9·6 (4·4–20·7)	<0·0001
Abnormality not tuberculosis	4315 (1·4%)	11 (2–77)	0·3 (0·0–1·8)	0·5 (0·1–3·7)	0·52	..	..	..	..
**WHO-estimated prevalence (per 100 000 people)**									
40–149	12 402 (3·9%)	8 (2–30)	0·1 (0·0–0·6)	0·2 (0·1–0·9)	0·034	4 (1–27)	0·6 (0·1–4·5)	1·2 (0·2–7·8)	0·876
150–349	39 287 (12·3%)	19 (12–31)	0·4 (0·2–0·6)	0·3 (0·2–0·6)	<0·0001	1 (0–8)	0·2 (0·0–1·4)	0·2 (0·0–1·6)	0·144
>350	267 294 (83·8%)	53 (47–59)	1·0	1·0	..	6 (4–9)	1·0	1·0	..
**Sputum culture testing**									
No	11 570 (3·6%)	39 (23–65)	1·0	1·0	..	3 (0–20)	1·0	1·0	..
Yes	307 413 (96·4%)	47 (42–53)	1·2 (0·7–2·1)	1·1 (0·6–1·9)	0·728	6 (4–8)	2·0 (0·3–14·7)	1·7 (0·3–11·0)	0·596

Data for screening before entry are for Jan 1, 2009, to Dec 31, 2012; those for the Enhanced Tuberculosis Surveillance system are for Jan 1, 2010, to Dec 31, 2013. The overall rate among those with assumed reactivation was 46 (95% CI 42–52) per 100 000 person-years; among assumed index cases it was 5 (4–8) per 100 000 person-years. Rows in which the IRRs=1·0 are the reference data. IRR=incidence rate ratio.
