# Supplementary material for: Tuberculosis in migrants moving from high-incidence to low-incidence countries: a population-based cohort study of 519 955 migrants screened before entry to England, Wales, and Northern Ireland
Source: Lancet. 2016 Nov 19;388(10059):2510–8. doi: 10.1016/S0140-6736(16)31008-X (PMC5121129; doi:10.1016/S0140-6736(16)31008-X)
Supplement: Supplementary appendix [file mmc1.pdf]

THE LANCET

Supplementary appendix

This appendix formed part of the original submission and has been peer reviewed. We post it as supplied by the authors.

Supplement to: Aldridge RW, Zenner, D White PJ, et al. Tuberculosis in migrants moving from high-incidence to low-incidence countries: a population-based cohort study of 519 955 migrants screened before entry to England, Wales, and Northern Ireland. *Lancet* 2016; published online Oct 11. http://dx.doi.org/10.1016/S0140-6736(16)31008-X.

Supporting information for Tuberculosis in migrants to the UK: a population-based cohort study of 519,955 migrants screened pre-entry, Aldridge et al.

1.1 Supplementary methods

1.1.1 Classification of radiographic findings

All visa applicants with radiological findings classified in group four "major findings sometimes seen in active tuberculosis" were required to undergo sputum testing for tuberculosis (Table 1). Individuals with chest radiographs classified as group three "minor findings occasionally associated with tuberculosis infection" were not mandatorily required to undergo sputum testing, but the panel physician responsible for the migrant screening (a medical doctor in charge of screening at an IOM clinic) was requested to consider sputum testing on a case-by-case basis. A radiologist and the panel physician both interpreted chest radiographs. For this analysis, major findings sometimes seen in active tuberculosis (or other conditions); or minor findings occasionally associated with tuberculosis infection and that panel physician determined on basis of clinical history and examination require dsputum sampling to rule out TB disease were classified in this analysis as compatible with TB. Applicants unwilling or unable to undergo radiographic screening were required to provide three consecutive daily sputum specimens that were tested in a designated laboratory for smear and culture.

Table 1. Classification of radiographic findings

**Minor Findings**	
1.1	Single fibrous streak/band/scar
1.2	Bony islets
2.1	Pleural capping with a smooth inferior border (<1cm thick at all points)
2.2	Unilateral or bilateral costophrenic angle blunting (below the horizontal)
2.3	Calcified nodule(s) in the hilum / mediastinum with no pulmonary granulomas
**Minor findings occasionally associated with tuberculosis infection**	
3.1	Solitary Granuloma (< 1 cm and of any lobe) with an unremarkable hilum
3.2	Solitary Granuloma (< 1 cm and of any lobe) with calcified / enlarged hilar lymph nodes
3.3	Single / Multiple calcified pulmonary nodules / micronodules with distinct borders
3.4	Calcified pleural lesions
3.5	Costophrenic Angle blunting (either side above the horizontal)
**Major findings sometimes seen in active tuberculosis (or other conditions)**	
4.0	Notable apical pleural capping (rough or ragged inferior border and/or ≥ 1cm thick at any point)
4.1	Apical fibronodular / fibrocalcific lesions or apical microcalcifications
4.2	Multiple / single pulmonary nodules / micronodules (noncalcified or poorly defined) 4.3 Isolated hilar or mediastinal mass/lymphadenopathy (non-calcified)
4.4	Single / multiple pulmonary nodules / masses ≥ 1 cm."
4.5	Non-calcified pleural fibrosis and / or effusion.
4.6	Interstitial fibrosis/ parenchymal lung disease/ acute pulmonary disease
4.7	Any cavitating lesion OR "fluffy" or "Soft" lesions felt likely to represent active TB

1.1.2 Duplicates

Individuals screened pre-entry may have multiple records on the IOM screening database. Duplicate records were analysed on the basis of whether they occurred within 12 months of each other or not. 12 months was chosen to distinguish duplicates on the basis that this period of time was long enough to capture individuals who were found to have tuberculosis on their first screen, and were undergoing repeat screening for visa clearance, but not too long a time period such that tuberculosis risk factors (including those listed in Table 4) would have changed significantly between screens.

Duplicates within 12 months:

Rules used to determine whether duplicate records within 12 months were included in the cohort or not are provided in Table 2. Individuals with a positive first and second screen would not have received a medical certificate of clearance, and therefore will never have been able to enter the UK and are excluded from this analysis. Migrants with a positive first screen and negative second screen are likely to have undergone treatment, been rescreened and found to be clear and will then have been able to enter UK. Therefore, the first screen is excluded, but the second included. Migrants with a negative first screen and positive second are unlikely to have entered the UK after the first screen due to short time period involved, and won't have received a medical certificate of clearance after the second screens. These records are likely to represent individuals who weren't able to get their visa processed within six months of the initial clearance and on repeat screening were found to be positive. They are therefore all excluded. Records negative on both screens are likely to be individuals who weren't able to get their visa processed within six months of the initial clearance and therefore the first is excluded, but the second is included.

Table 2. Rules for dealing with duplicate screens less than 12 months apart.

		**First screen TB result**	
		+ve	-ve
**Last screen TB result**	+ve	Exclude all	Exclude all
	-ve	1st screen: Exclude<br>2nd screen: Include	1st screen: Exclude<br>2nd screen: Include

Duplicates greater than 12 months apart:

Rules used to determine whether duplicate records occurring more than 12 months apart were included in the cohort analysis are provided in Table 3. Individuals with a positive first and second screen will not have been given a medical certificate of clearance, and therefore will never have been able to enter the UK. All these records are therefore excluded. Records with a positive first screen, but negative second screen are likely to represent migrants reapplying after treatment because their initial screen was positive. Therefore the first screen is excluded but the second included. Migrants with the negative first screen, but positive second screen are allowed to enter the cohort after the first screen but are censored one month prior to the second screen, and the second screen is excluded. Records with negative first and second screen could represent a variety of different scenarios, including: 1) individuals that have visited the UK and are reapplying for a new visa having returned to their country of origin after their initial visa ran out; and 2) individuals reapplying because their medical clearance ran out in the time they were able to get their visa application processed (clearance certificates are only valid for 6 months). These individuals are therefore allowed to enter the cohort after each screen, but are censored one month prior to the next screen to allow for return to the country of origin.

Table 3. Rules for dealing with duplicate screens greater than 12 months apart.

		**First screen TB result**	
		+ve	-ve
**Last screen TB result**	+ve	Exclude all	1st: Include and censor at 1 month prior to 2nd screen<br>2nd: Exclude
	-ve	Exclude all duplicates apart from the last screen	1st: include and censor at 1 month prior to 2nd screen

			2nd: Include

1.1.3 Sample size

Before access to the IOM database was granted, it was assumed there would be 350,000 individuals eligible for this study with an estimated length of follow up 1,040,000 person years. It was estimated that incidence rates in individuals from high, medium and low risk countries (defined as 300, 150 and 40 cases per 100k person years respectively) would be calculated with the following confidence intervals: 300: 95% CIs 294-306; 150: 95% CIs 142-158; and 40: 95% CIs 32-48.

1.1.4 Variable classifications and definitions.

Age and sex were considered a-priori as confounding variables. Risk factor variables from the IOM dataset included: visa category, contact with a case of tuberculosis, CXR classification at pre-entry screening, whether a migrant was screened at a clinic where culture testing of sputum samples was performed, and WHO prevalence estimates of tuberculosis in the country of origin. WHO prevalence was used to stratify countries estimates, rather than incidence estimates, for consistency with previously published work. (1)

Table 4. Data sources of variables, methods of assessment, details of subgroups

Variable	Data Source	Methods of assessment	Subgroups chosen for analysis
Age	IOM	From visa application data	0-15; 16-44; 45-64; >65
Sex	IOM	From visa application data	Male; Female
Close or household contact with a case of TB	IOM	Self report to panel physician screening applicant	Yes; No. Where null assumed to be No
Visa Type	IOM	From visa application data	• Student - All students who receive (or do not received) a scholarship;<br>• Settlement and Dependent - Those applicants who are settling permanently in the UK, as well as spouses, partners/civil partners, fiancées, fiancés, proposed partners/civil partners, children, other dependant relatives;<br>• Work - This type of visa is intended for work related visas, including work requiring a permit, and the highly skilled migrant program. This category EXCLUDES the working holiday maker category, which has been assigned a separate category (see below);<br>• Working Holiday Maker - Applicants going to the UK on the working holiday maker program;<br>• Family Reunion - Type of settlement that allows the family members of a recognized refugee to join him/her in the UK;<br>• Other - this category is used when the applicant's visa category is not readily identified.
CXR	IOM	CXR classification chosen by panel physician after double reading by radiologist.	• No abnormality – no findings on CXR;<br>• Compatible with TB – As detailed in Table 1 of appendix - major findings sometimes seen in active tuberculosis (or other conditions); or Minor findings occasionally associated with tuberculosis infection and that panel physician determines on basis of clinical history and examination require sputum sampling to rule out TB disease.<br>• Abnormality not TB – Finding on CXR that is not normal, but does not consistent with TB

Prevalence of TB in country of migration	WHO	WHO modelling data taken from http://www.who.int/tb/publications/global_report/en/	40-149; 150-349; 350+
Year of examination	IOM	Based on IOM data and date of screening recorded by panel physician.	2005 ;2006; 2007; 2008; 2009; 2010; 2011; 2012; 2013
Country of screening	IOM	From screening location recorded in IOM dataset	Burkina Faso; Bangladesh; Cambodia; Cote D'Ivoire; Eritrea; Ghana; Kenya; Laos; Niger; Pakistan; Sudan; Somalia; Togo; Tanzania; Thailand
Sputum culture testing	IOM	From IOM dataset based on dates of the roll out of culture testing according to UK technical instructions.	Yes; No

1.1.5 Molecular epidemiology

Assumed reactivation cases were examined to distinguish disease that is potentially avoidable through treatment of latent infection. First in cluster cases were examined as these cases are involved in onward chains of tuberculosis transmission after arrival in the UK and therefore preventing these has the potential to avert additional cases of locally acquired disease.

1.1.6 Statistical analysis

Baseline descriptive statistics of the cohort were provided using simple counts and proportions. To account for the uncertainty of death, migration to Scotland and emigration, multiple imputation was performed. External data were used to inform the imputation models as described in the following two sections. Ten imputed datasets were created.

Population attributable fraction (PAF) was calculated using the formula in Equation 1. Individual imputed datasets were analysed using Poisson regression, a suitable method for modelling rare event data, and estimates of crude and adjusted incidence rates for the primary and secondary outcomes were calculated. A multivariable Poisson regression model was used to identify risk factors for the primary outcomes. All results were adjusted for clustering by individual, to take account of repeated entries by migrants into the cohort. The results of the analyses from individual imputed datasets were then combined using Rubin’s rules; these appropriately account for uncertainty in the imputed information. Final results were presented as incidence rate per 100,000 person years at risk, incidence rate ratios, 95% confidence intervals and p-values.

Equation 1. Formula used for calculation of Population attributable fraction (PAF).(2)

$$\boldsymbol{PAF = \frac{p * (Relative\ risk - 1)}{p * (Relative\ risk - 1) + 1}}$$

p=proportion of the total population exposed to the risk factor.

1.1.7 Migration out of the country

No data were available to be able to determine how long each individual migrant screened pre-entry was able to stay in EWNI. It was therefore necessary to impute data on length of stay, and hence time at risk of being notified as a case of tuberculosis was based on visa category that was collected at the time of pre-entry screening. Lengths of stay were imputed using data on UK entry clearance visas, which details length of stay by type of visa and year of issue.(3) These data have high coverage for all visas issued outside the UK to non-EEA nationals who are subject to immigration control and require a visa to enter the UK.

These data include the number of visas issued, by year and by duration of stay (0-3mths, 3-6mths, 6mths-1yr, 1-2yrs, 2-3yrs, 3-4yrs, 4+yr). For the purposes of imputation, visas were grouped into four categories: total; work; study; or student visitor (which relates to individuals attending short courses for a period of less than one year). The "total" category excluded visitor and transit visas, but included all other categories including work, both types of study, family reunion, dependant's joining or accompanying individuals already in the UK, and other rarer visa types. The proportion of study and student visas that were issued for short courses is detailed in Table 5.

Table 5. Percentage of study and student visas that are for short courses

Year	Percentage
2005	8
2006	10
2007	13
2008	15
2009	11
2010	15
2011	19
2012	25
2013	26

IOM pre-entry screening data had the following visa categories (Table 4 provides definitions for each): family reunion, settlement, work, working holiday, students and other. Table 6 shows how IOM visa categories were matched to visa duration data. Imputation assumed that migrants remained in England, Wales and Northern Ireland (EWNI) for the duration of the visa issued.

Table 6. Rules for imputing visa duration

IOM visa category	Visa duration data category	Imputation rule
Family reunion	Included in "total"	Assume these stay for the duration of the cohort analysis.
Settlement	Included in "total"	Assume these stay for the duration of the cohort analysis.
Students	Study and short study	(i) Select long/short course according to probabilities in Table 4.<br>(ii) Then impute from either the study or short-course study distribution.
Work	Work	Impute from work distribution.
Working holiday	Work	Impute from work distribution, restricting to categories < 2yrs (working holidays are maximum 2yrs)
Other	Included in "total"	Impute from overall distribution

The distributions of visa duration over time provide data for the imputation of length of stay. Figures 1-4 show the distribution of the duration of visas issued over time by visa category, from 2005 to 2012. Overall fewer long-

term (4+yrs) visas were issued from 2009, with a corresponding increase short-term (3mths-1yr) and mid-long-term visas (3-4yrs). For work visas, fewer long-term visas (4+yrs) have been issued since 2009. The duration of study visas has stayed broadly similar over time, whereas the short course study visas for 0-3mths were not issued in recent years.

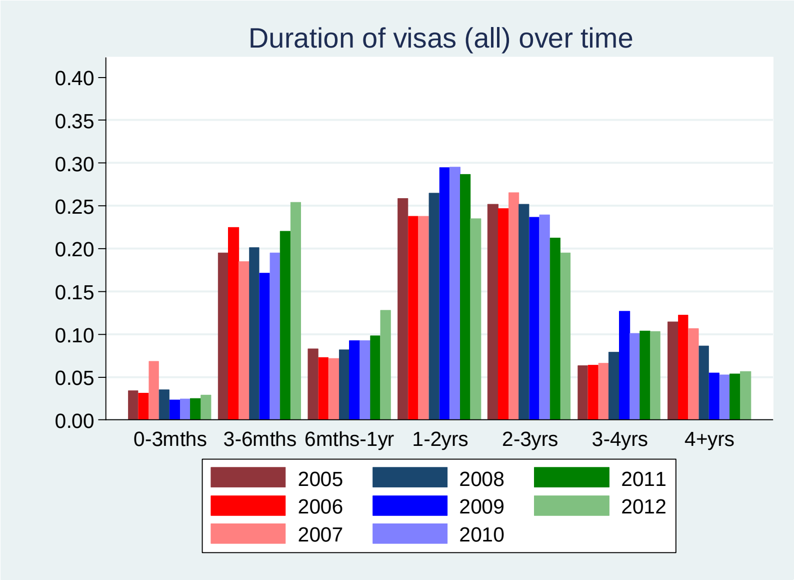

Figure 1. **Changes in the distribution of all visa durations over time.**

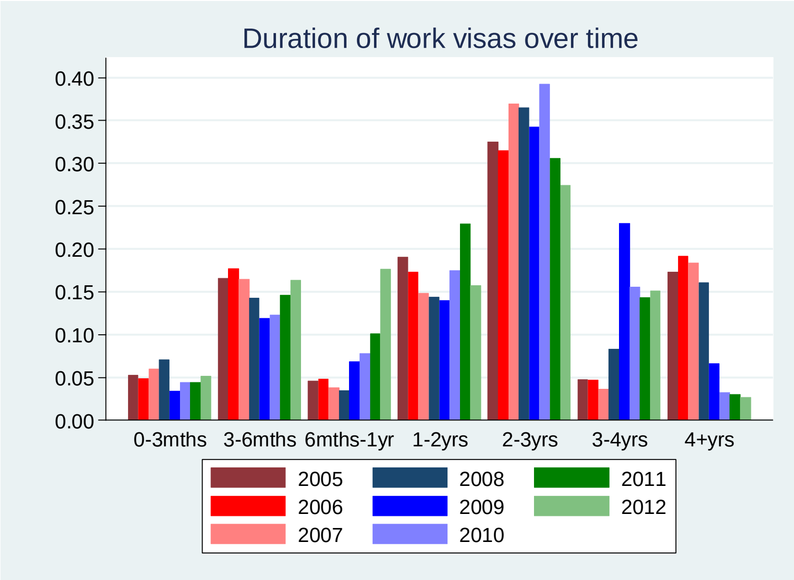

Figure 2. **Changes in distribution of work visa duration over time.**

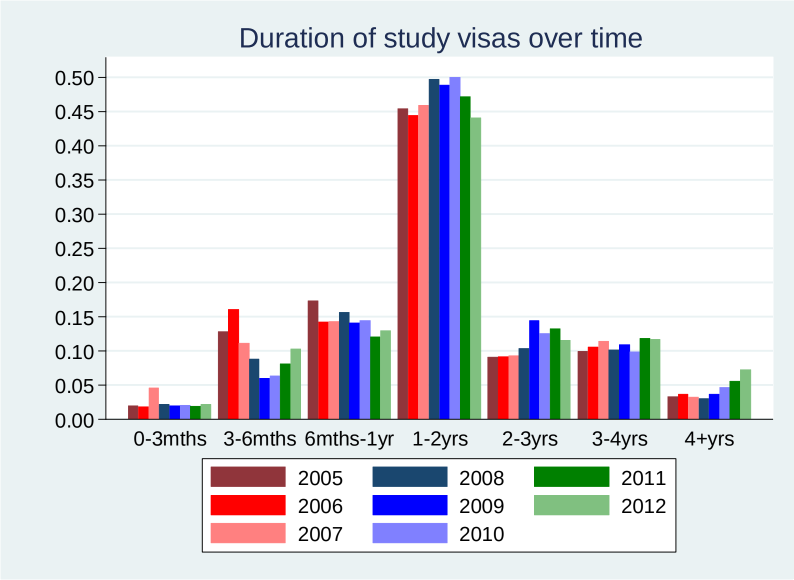

Figure 3. Changes in distribution of study visa duration over time

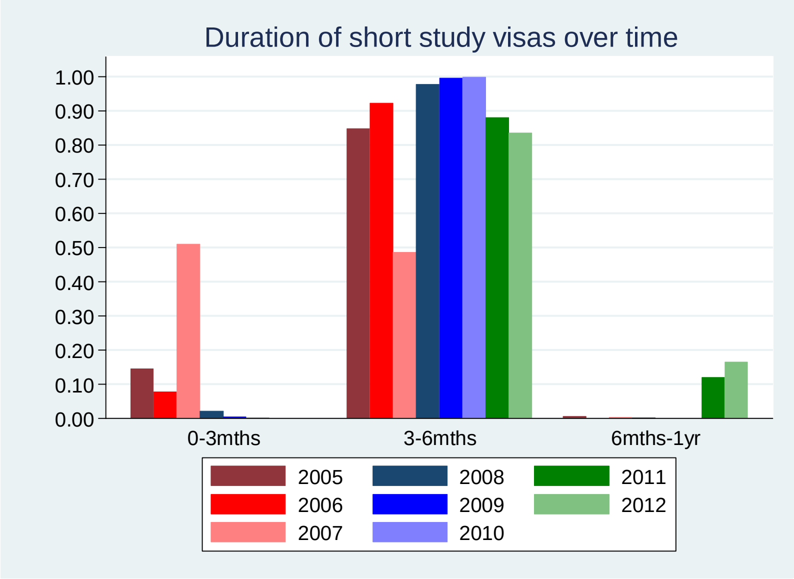

Figure 4. Changes in distribution of short study visa duration over time

The distributions in the visa issue data provide an imputed visa category for length of stay e.g. 1-2 yrs. To impute the exact duration of stay, a time for each migrant within a visa category was chosen by linear interpolation. For example, if a random uniform variable used for this imputation lands 25% of the way between the cumulative probabilities of categories 1-2yrs, the duration that lies 25% of the way between 2yrs and 1yr was used, which is equal to 1.25yrs.

1.1.8 Estimating the rate of application for extension to student visa

For student visas, a large number of applicants get visa extensions and for this group we therefore made some additional assumptions to extend their length of stay. Data are available on the number of applications for extensions to student visas during 2010-2014.(4,5) Therefore, to estimate a rate of application (during this period of time), we need to obtain an appropriate denominator: the total number of students who were in the UK, and might plausibly be applying for an extension. We have attempted to estimate the number of students in the UK at this time whose visas had just expired and thus might apply for an extension.

We have calculated a distribution for the length of student stay in the UK for students arriving during years 2005-2012. We did not have data for years 2013-2014, thus for this calculation we assumed these numbers remained stable from 2012-2014.

Applying these numbers to the numbers of student entries (Table 7) gives a distribution of number of students by planned year of leaving the UK (according to visa expiry date) (Table 8). This is assumed to be the year they apply for a visa extension. Note that Table 8 does not take account of students entering prior to 2005 who have already remained for 5 or more years as we did not have the data to allow us to do this, however, this accounted for a very small number of cases given screening numbers in 2005.

Putting the data from Table 8 together with the number of applications for extensions made, we can estimate the rate of visa extension application as shown in Table 9.

Table 7. Number of UK Entry clearance visas issued overseas, 2005 to 2013, for study

Year	Study (thousands)	Percentage of student visas that are short courses
2005	191,600	8
2006	211,100	10
2007	213,000	13
2008	231,900	15
2009	303,300	11
2010	285,500	15
2011	261,900	19
2012	209,800	25
2013	218,600	26

Table 8. Number of students in the UK, by year of visa expiry

Year current visa expires	Number of students in UK
2010	194,870
2011	232,279
2012	228,753
2013	206,800
2014	169,796

Table 9. Estimated rates of application for student visa extension

Year	Number of extension Applications	Students with visas expiring this year	Proportion who apply for extension
2010	84,230	194,870	0.43
2011	107,270	232,279	0.46

2012	79,722	228,753	0.35
2013	108,029	206,800	0.52
2014	73,041	169,796	0.43

1.1.9 Estimating the rate of success from applications for extension to student visa

Although the rate of application does not appear to vary widely over time, the rate of applications being successful is likely to have changed over time.(5) Table 10 shows the decisions on applications for an extension of stay by category.

Table 10. Estimated rates of success in applications for visa extension

Year	Decisions regarding student visa extension*	Number of grants	Proportion whose application was successful
2008	139,141	132,366	0.95
2009	149,391	129,579	0.87
2010	164,072	140,151	0.85
2011	136,279	119,303	0.88
2012	100,296	87,073	0.87

(*Decisions on applications for an extension of stay by category)

1.1.10 Estimating the duration of stay probabilities, allowing up to two extensions to student visas

Using these results we then estimated the cumulative probabilities of staying up to 0.25, 0.5, 1, 2, 3, 4, 5+ years, incorporating the possibility of extending the visa one or two times. We assume that the second and third visas are all of the same duration as the first.

Table 11 shows the duration of stay in the UK under 0, 1, and 2 visa extensions, by initial visa length, assuming all visas for an individual are for the same length of time. Table 11 also shows the probabilities of doing so. ($P_{0.25}$, $P_{0.25}$, $P_{0.25}$,… are the probabilities of each visa duration, as calculated previously; Pe_0, Pe_1, Pe_2, are the rates of visa extension applications 0, 1, and 2 years after UK entry for that individual; Ps_0, Ps_1, Ps_2, are the rates of application success in years 0, 1, and 2 after UK entry for that individual.)

Table 11. Duration of stay under 0, 1, and 2 extensions with probabilities of doing so

Max length Initial visa		Year student who enters UK in year Y leaves if…					
	Prob	Prob(no extension)	…No extension	Prob(1 extension)	…1 extension	Prob(2 extensions)	…2 extensions
0.25	$P_{0.25}$	$(1-Pe_0) + Pe_0 (1-Ps_0)$	Y+0.25	$Pe_0*Ps_0*\{(1-Pe_0) + Pe_0*(1-Ps_0)\}$	Y+0.5	$Pe_0*Ps_0*Pe_0*Ps_0$	Y+0.75

0.5	$P_{0.5}$	$(1-Pe_0) + Pe_0 (1-Ps_0)$	Y+0.5	$Pe_0*Ps_0*\{(1-Pe_1) + Pe_1*(1-Ps_1)\}$	Y+1	$Pe_0*Ps_0*Pe_1*Ps_1$	Y+1.5
1	P_1	$(1-Pe_1) + Pe_1 (1-Ps_1)$	Y+1	$Pe_1*Ps_1*\{(1-Pe_2) + Pe_2*(1-Ps_2)\}$	Y+2	$Pe_1*Ps_1*Pe_2*Ps_2$	Y+3
2	P_2	$(1-Pe_2) + Pe_2 (1-Ps_2)$	Y+2	$Pe_2*Ps_2*\{(1-Pe_4) + Pe_4*(1-Ps_4)\}$	Y+4 (&*)	$Pe_2*Ps_2*Pe_4*Ps_4$	Y+6*
3	P_3	$(1-Pe_3) + Pe_3 (1-Ps_3)$	Y+3	$Pe_3*Ps_3*\{(1-Pe_6) + Pe_6*(1-Ps_6)\}$	Y+6 (&*)	$Pe_3*Ps_3*Pe_6*Ps_6$	Y+9
4	P_4	$(1-Pe_4) + Pe_4 (1-Ps_4)$	Y+4	$Pe_4*Ps_4*\{(1-Pe_8) + Pe_8*(1-Ps_8)\}$	Y+8*	$Pe_4*Ps_4*Pe_8*Ps_8$	Y+12
5+	P_5	$(1-Pe_5) + Pe_5 (1-Ps_5)$	>Y+5	$Pe_5*Ps_5*\{(1-Pe_{10}) + Pe_{10}*(1-Ps_{10})\}$	>Y+10		>Y+20

* To avoid jumps in student distribution, these people were split evenly between the min and max years allowed in that visa range

Table 12a shows the different permutations of visas that end up with an individual having each category of duration of stay. Table 12b shows the same information in probabilities.

Table 12a. Different ways of ending up with each category of duration of stay, with up to 2 visa extensions allowed

Total length of stay	Ways in which this length of stay can happen
Y + 0.25	No extensions (0.25)
Y + 0.5	No extensions (0.5); 1 extension (0.25x2)
Y + 1	No extensions (1); 1 extension (0.5x2); 2 extensions (0.25x3)
Y + 2	No extensions (2); 1 extension (1x2); 2 extensions (0.5x3)
Y + 3	No extensions (3); 1 extension (2x2) (half of these); 2 extensions (1x3)
Y + 4	No extensions (4); 1 extension (2x2) (half of these); 2 extensions (2x3) (third of these)
>=Y+5	Everything else

Table 12b. Probabilities of different ways of ending up with each category of duration of stay, with up to 2 visa extensions allowed

Total length of stay	Ways in which this length of stay can happen in terms of probabilities above
Y + 0.25	$P_{0.25}*\{(1-Pe_0) + Pe_0 (1-Ps_0)\}$

Y + 0.5	$P_{0.5}$ *{(1-Pe_0) + Pe_0 (1-Ps_0)} + $P_{0.25}$*Pe_0*Ps_0*{(1-Pe_0) + Pe_0*(1-Ps_0)}
Y + 1	P_1 *{(1-Pe_1) + Pe_1 (1-Ps_1)} + $P_{0.5}$*Pe_0*Ps_0*{(1-Pe_1) + Pe_1*(1-Ps_1)} + $P_{0.25}$*Pe_0*Ps_0*Pe_0*Ps_0
Y + 2	P_2 *{(1-Pe_2) + Pe_2 (1-Ps_2)} + P_1*Pe_1*Ps_1*{(1-Pe_2) + Pe_2*(1-Ps_2)} + $P_{0.5}$*Pe_0*Ps_0*Pe_1*Ps_1
Y + 3	P_3 *{(1-Pe_3) + Pe_3 (1-Ps_3)} + (P_2/2)*Pe_2*Ps_2*{(1-Pe_4) + Pe_4*(1-Ps_4)} + P_1*Pe_1*Ps_1*Pe_2*Ps_2
Y + 4	P_4 *{(1-Pe_4) + Pe_4 (1-Ps_4)} + (P_2/2)*Pe_2*Ps_2*{(1-Pe_4) + Pe_4*(1-Ps_4)} + (P_2/3)*Pe_2*Ps_2*Pe_4*Ps_4
>=Y+5	Everything else (i.e. 1 – other probabilities)

1.1.11 Death:

To estimate survivor functions, Kaplan-Meier product-limit survival probabilities were calculated using the survival rates from 2009 data for England and Wales (Figure 5). These data were created by the Cancer Research UK Cancer Survival Group at LSHTM, and is based upon mortality data provided by ONS.(6)

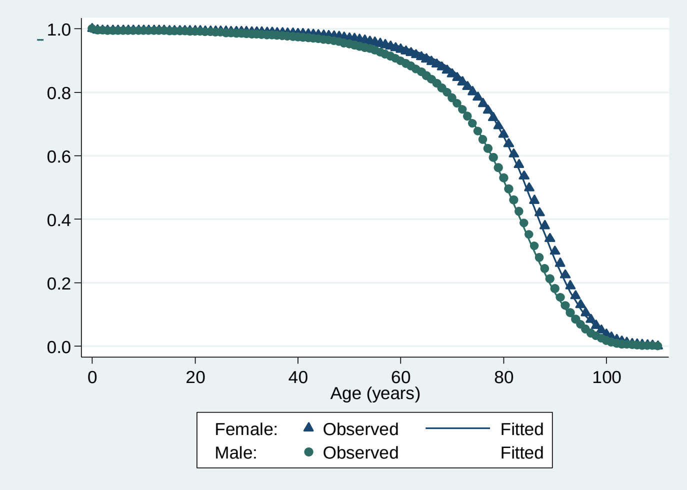

Figure 5. Survival probabilities calculated from England and Wales 2009 data, with fitted line

To model these survival probabilities, the logit transformation of the survival probability was created, and then modelled using linear regression as a cubic function of exponentiated age. The logit transformation provides a fit to the observed data with a smoother and more stable gradient. The exponential transformation of age enables a better fit for older ages, which is where the survival changes rapidly, and is therefore important to represent accurately. The fitted model is given in Equations 2 and 3.

$$\ln\left(\frac{p}{1-p}\right) = 9.231431 - 5.106226 \times \mathrm{e}^{\mathrm{age}} + 2.250698 \times (\mathrm{e}^{\mathrm{age}})^2 - 0.7572033 \times (\mathrm{e}^{\mathrm{age}})^3$$

Equation 2. For men:

$$\ln\left(\frac{p}{1-p}\right) = 11.45322 - 8.980079 \times \mathrm{e}^{\mathrm{age}} + 4.07654 \times (\mathrm{e}^{\mathrm{age}})^2 - 1.049887 \times (\mathrm{e}^{\mathrm{age}})^3$$

Equation 3. For women:

As illustrated in Figure 5 this function provides a good fit to the observed data. In order to draw from this distribution it was necessary to be able to calculate the age corresponding to a particular fitted value of the survival probability. This was done by solving the cubic equation created by entering a specific value for p in the two equations above. Figure 6 shows the ages calculated from survival probabilities across the interval 0-1, with the fitted survival probabilities generated from the model above overlaid.

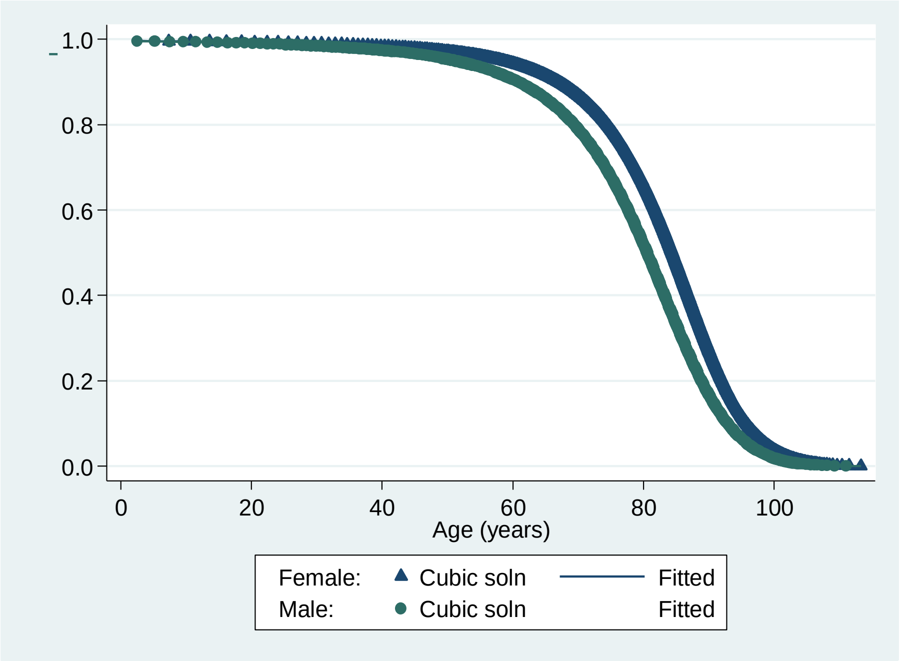

Figure 6. Ages calculated from survival probabilities, with fitted line from

Age at of death for someone entering the cohort at age 0 was imputed by:

(i) Drawing a random value from the standard uniform distribution, U~[0,1].
(ii) Setting the predicted age of death to be the age that corresponds to the survival probability U.

Age at death for someone who is already aged x years, death was imputed by:

(i) Drawing a random value from the standard uniform distribution, U~[0,1].
(ii) Calculated the survival probability for age x, S_x
(iii) Set the predicted age at death to be the age that corresponds to the survival probability U x S_x.

1.1.12 Migrants to Scotland:

Personal identifiable information was not available for tuberculosis notifications in Scotland. Therefore migrants to Scotland could not be identified as incident cases for any of the primary or secondary outcomes. It was therefore necessary to specify that a proportion of migrants given a medical certificate of clearance would not enter the cohort. Using data on long-term international migration produced by ONS, it was estimated that 7.3% of migrants entering the UK between 2006 and 2012 would be resident in Scotland.(7) Therefore, for each imputation, 92.7% of migrants issued with a medical certificate of clearance were randomly selected (from a uniform distribution) to enter the cohort in England, Wales, and Northern Ireland (EWNI). This issue is examined further in a sensitivity analysis.

1.1.13 Sensitivity analyses:

The assumptions made in this analysis were examined by several sensitivity analyses. The rules used to de-duplicate the cohort were examined in order to assess changes in the primary outcomes. In a sensitivity analysis, all individuals apart from those with a positive screen were allowed to enter the cohort, but censored one month prior to re-screening. This analysis will provide a more conservative estimate for person time at risk, and therefore reduce the incidence rates to a lower estimate.

Migration out of EWNI was examined. In the first analysis, all migrants were assumed to stay for one and a half years, providing a lower estimate of person time at risk. In a second sensitivity

analysis, all migrants were assumed to stay until the end of the study period of 31st December 2013. This is the more conservative assumption, and whilst it unrealistically inflates the denominator, it will provide a lower bound for the estimates of incidence.

Death was also examined by assuming there were no deaths in the cohort. This is the most conservative assumption and unrealistically inflates the denominator, but also provides a lower bound for the estimates of incidence.

Varying the definition of how incident and prevalent cases were distinguished (i.e. the 90 day cut off between pre-entry screening and notification) was examined. For the main analysis, it was assumed that cases notified within 90 days of pre-entry screening were ‘missed’ prevalent and not incident cases. The impact of varying this definition (from 90 to 180 days) on the outcomes was assessed.

The time period used for strain typing data was examined to see how this affected the incidence rates of likely reactivation and index cases. Finally, we examined the impact of assuming that the clustering in untyped cases would be similar to that in typed cases to estimate a crude upper bound of assumed reactivation cases.

1.2 Supplementary results

Table 13 describes the very low levels of missing data across the matching variables and Table 14 shows the baseline characteristics, univariate and multivariate analysis of incidence rates for extra-pulmonary tuberculosis and bacteriological confirmed extra-pulmonary tuberculosis in migrants screened pre-entry.

Table 13. Description of missing data for linkage variables in ETS and IOM pre-entry screening dataset

	ETS Missing data		IOM Missing data	
**Variable**	**N**	**%**	**N**	**%**
First name	0	0%	13078	2%
Surname	0	0%	4824	1%
Date of Birth	32	0%	910	0%
Sex	186	0%	786	0%
Nationality	1823	2%	796	0%

Numbers of entries in data sets: ETS: 83781; IOM: 640808.

Table 14. **Baseline characteristics, univariate and multivariate analysis of incidence rates for extra-pulmonary tuberculosis (all forms including clinically diagnosed cases) and bacteriological confirmed extra-pulmonary tuberculosis in migrants screened pre-entry (2006-2012) and notified in ETS (2006-2013)**

		All extra-pulmonary cases of tuberculosis				Bacteriological confirmed extra-pulmonary tuberculosis			
Risk Factor	Migrants contributing (%)	Rate per 100,000 person years (95% CI)	Univariable IRR (95% CI)	Multivariable IRR (95%CI)	p-value	Rate per 100,000 person years (95% CI)	Univariable IRR (95% CI)	Multivariable IRR (95%CI)	p-value
All	519955 (100%)	84 (79, 89)	-	-	-	53 (49, 57)	-	-	-
Age									
0-15	15468 (3%)	54 (38, 78)	0.6 (0.4, 0.9)	0.7 (0.5, 1)	0.062	21 (11, 37)	0.4 (0.2, 0.7)	0.4 (0.2, 0.8)	0.005
16-44	490806 (94.4%)	85 (80, 90)	1.0	1.0		54 (50, 59)	1.0	1.0	
45-64	11243 (2.2%)	97 (69, 136)	1.1 (0.8, 1.6)	1.3 (0.9, 1.8)	0.167	61 (40, 94)	1.1 (0.7, 1.7)	1.3 (0.9, 2.1)	0.201
65+	2438 (0.5%)	92 (43, 194)	1.1 (0.5, 2.3)	1 (0.5, 2.1)	0.936	39 (13, 122)	0.7 (0.2, 2.2)	0.7 (0.2, 2.1)	0.514
Sex									
Female	173116 (33.3%)	72 (66, 80)	1.0	1.0		46 (40, 52)	1.0	1.0	
Male	346839 (66.7%)	93 (86, 100)	1.3 (1.1, 1.4)	1 (0.9, 1.2)	0.565	59 (53, 64)	1.3 (1.1, 1.5)	1 (0.8, 1.2)	0.792
Contact with case TB									
No	518735 (99.8%)	84 (79, 89)	1.0			53 (49, 57)	1.0	1.0	
Yes	1220 (0.2%)	124 (46, 329)	1.5 (0.5, 3.9)	1.6 (0.6, 4.3)	0.344	93 (30, 287)	1.7 (0.6, 5.4)	2 (0.6, 6.2)	0.247
Visa									
Students	307127 (59.1%)	93 (85, 101)	1.0	1.0		61 (55, 68)	1.0	1.0	

Settlement and Dependents	159986 (30.8%)	72 (65, 79)	0.8 (0.7, 0.9)	0.8 (0.7, 1)	0.014	44 (39, 49)	0.7 (0.6, 0.8)	0.8 (0.6, 0.9)	0.006
Work	21140 (4.1%)	107 (81, 143)	1.2 (0.9, 1.6)	1.1 (0.8, 1.5)	0.476	62 (42, 90)	1 (0.7, 1.5)	1 (0.7, 1.4)	0.908
Working Holiday Maker	17526 (3.4%)	124 (77, 199)	1.3 (0.8, 2.2)	1.8 (1.1, 2.8)	0.021	80 (44, 145)	1.3 (0.7, 2.4)	1.8 (1, 3.2)	0.060
Family Reunion	3989 (0.8%)	181 (127, 259)	1.9 (1.3, 2.8)	2.8 (1.9, 4.1)	<0.001	97 (59, 158)	1.6 (1, 2.6)	2.4 (1.4, 4.1)	0.001
Other	10187 (2%)	53 (28, 102)	0.6 (0.3, 1.1)	1 (0.5, 1.8)	0.915	36 (16, 79)	0.6 (0.3, 1.3)	1 (0.5, 2.2)	0.98
CXR									
No abnormality	489733 (94.2%)	81 (76, 87)	1.0	1.0		52 (48, 56)	1.0	1.0	
TB suspected	21862 (4.2%)	154 (125, 191)	1.9 (1.5, 2.3)	1.9 (1.5, 2.4)	<0.001	90 (69, 119)	1.7 (1.3, 2.3)	1.8 (1.3, 2.4)	<0.001
Abnormality not TB	8360 (1.6%)	55 (31, 96)	0.7 (0.4, 1.2)	1 (0.5, 1.7)	0.902	36 (18, 73)	0.7 (0.4, 1.4)	1.1 (0.5, 2.1)	0.884
WHO category									
40-149	29143 (5.6%)	18 (10, 33)	0.2 (0.1, 0.4)	0.2 (0.1, 0.3)	<0.001	10 (5, 22)	0.2 (0.1, 0.4)	0.2 (0.1, 0.4)	<0.001
150-349	75294 (14.5%)	57 (47, 68)	0.6 (0.5, 0.7)	0.5 (0.4, 0.7)	<0.001	35 (28, 44)	0.6 (0.5, 0.7)	0.5 (0.4, 0.7)	<0.001
350+	415518 (79.9%)	94 (88, 100)	1.0	1.0		60 (55, 65)	1.0	1.0	
Sputum culture testing									
No	179935 (34.6%)	87 (79, 95)	1.0	1.0		53 (47, 59)	1.0	1.0	
Yes	340020 (65.4%)	82 (76, 89)	0.9 (0.8, 1.1)	0.9 (0.8, 1)	0.044	53 (48, 59)	1 (0.9, 1.2)	0.9 (0.8, 1.1)	0.293

A series of sensitivity analyses were carried out to examine the effect varying the assumptions made in the cohort analyses. Varying the definition of a prevalent case from zero to 180 days after issuing a medical certificate of clearance (from the baseline assumption of 90 days) had little impact on the estimates of the incidence of all tuberculosis cases, bacteriologically confirmed cases of pulmonary tuberculosis, assumed index case or assumed reactivation (Figure 7). Incidence rates increased slightly when reducing the prevalent case definition to zero days and when including only cases screened using the culture confirmation protocol, but reduced when increasing the prevalent case definition to 180 days. The 95% confidence intervals for the incidence rates overlapped for each sensitivity analysis, providing no evidence that any of these estimates were statistically different to the baseline scenario.

Varying these same assumptions in multivariable analyses to look at risk factors for the primary outcomes also had very little effect on the results (Figures 8-11). There was strong evidence in each sensitivity analysis that having a chest radiograph classified as suspected tuberculosis was associated with an increased risk of being the first case in a cluster. In the baseline analysis this was the only group associated with an increased risk, and this remained the case in all scenarios in the sensitivity analysis, but due to the fact that there were no first cases in a cluster for some subcategories, several variables had null results in sensitivity analysis (e.g. work and other visa categories, and WHO prevalence countries between 40 and 149). In the sensitivity analysis for assumed reactivation as the primary outcome, there remained strong evidence that coming from a country with WHO prevalence of less than 350 per 100,000 population resulted in a reduced risk of assumed reactivation. All other results in the sensitivity analysis for this outcome remained consistent with the baseline assumptions.

Varying the assumptions relating to the definition of a prevalent case, de-duplication in the migrant screening dataset, deaths, migration to Scotland made little difference to crude incidence rates and the multivariable analyses (Supplementary appendix Figures 7-10). Assuming that migrants cleared for entry remained in the cohort until the end of the study period reduced the rates of disease for primary and secondary outcomes. In the multivariable sensitivity analysis this assumption increased the risk of all forms of tuberculosis and bacteriologically confirmed pulmonary tuberculosis for settlement and dependant and family reunion visas. Assuming all migrants remain in the cohort until the end of the study increases the risk for settlement and dependant and family reunion visas as this assumption reduces the risk in the baseline comparator group (students) by increasing their person time at risk. Settlement and dependant and family reunion visas, however, were already assumed to stay in the cohort for the duration of the study, and therefore their rates of disease remain static in this sensitivity analysis. In the multivariable analysis therefore the risk in students goes down under this assumption compared to the Settlement and dependant and family reunion visas, explaining this otherwise unusual finding. The risk was reduced for working holiday maker visas and sputum culture testing was no longer associated with a reduced risk of developing tuberculosis for the primary outcomes. Assuming that migrants remained in the cohort for a minimum of 12 months increased the rates of disease for primary outcomes (as a result of reducing the overall person time at risk – the denominator – compared to the numerator), and had similar effects on the multivariable analysis to assuming all migrants remained in the cohort until the end of the study.

Data used for linkage by strain typing are subject to statistical censoring due to limited time of follow-up. Sensitivity analysis examined whether the amount of strain typing data available affected the estimated incidence of reactivation and index cases: increasing the time window from 1 year of strain typing data (2010) to 3 (2010-2012) increased the proportion of cases clustered from 48.3% (95%CI:46.6-50.0) to 53.1% (95%CI:52.3-54.0); this did not increase significantly with an additional year's data (53.5%; 95%CI:52.8-54.3; Supplementary appendix Figure 12).

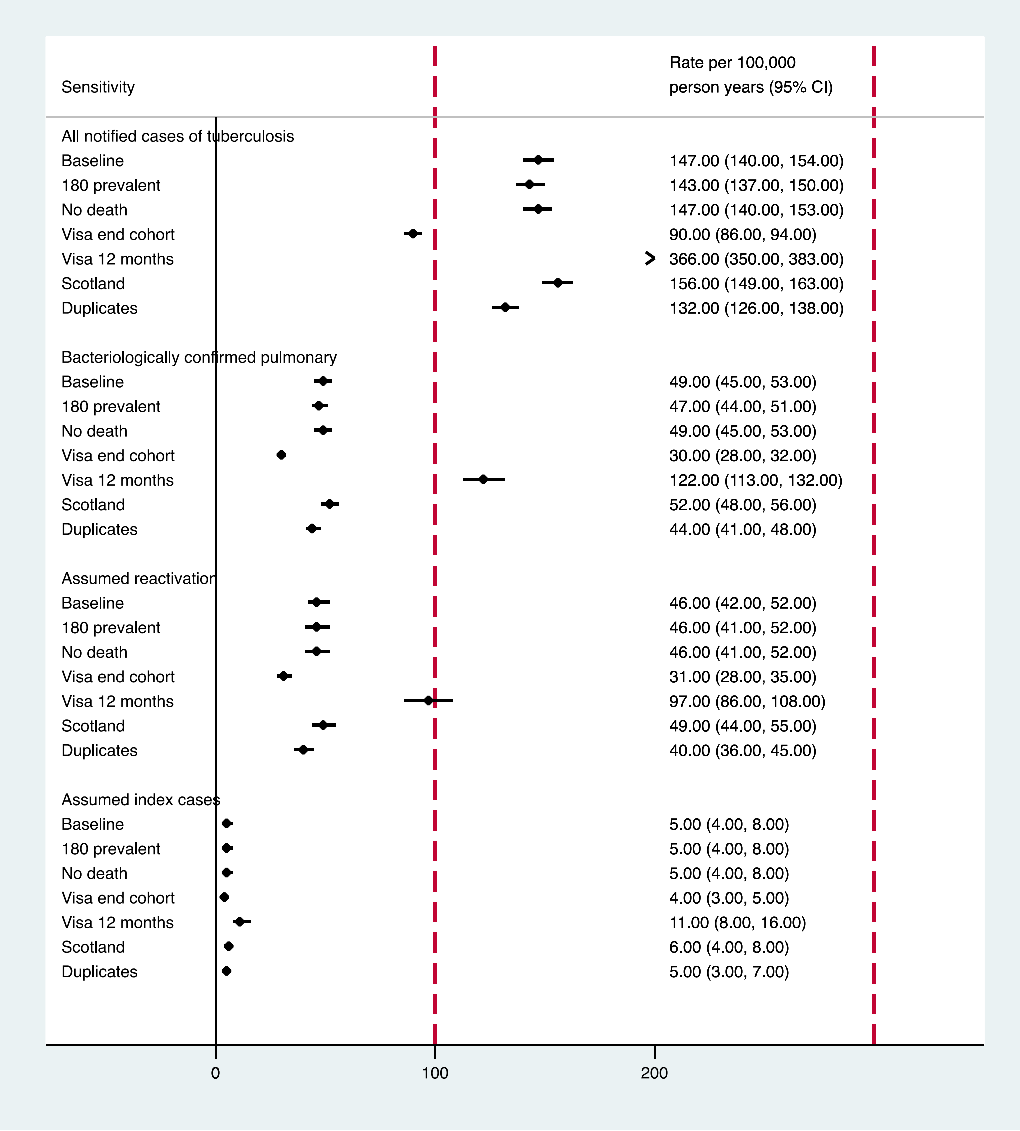

Figure 7. **Sensitivity analysis of estimates of incidence rates for primary and secondary outcomes under different model assumptions.**

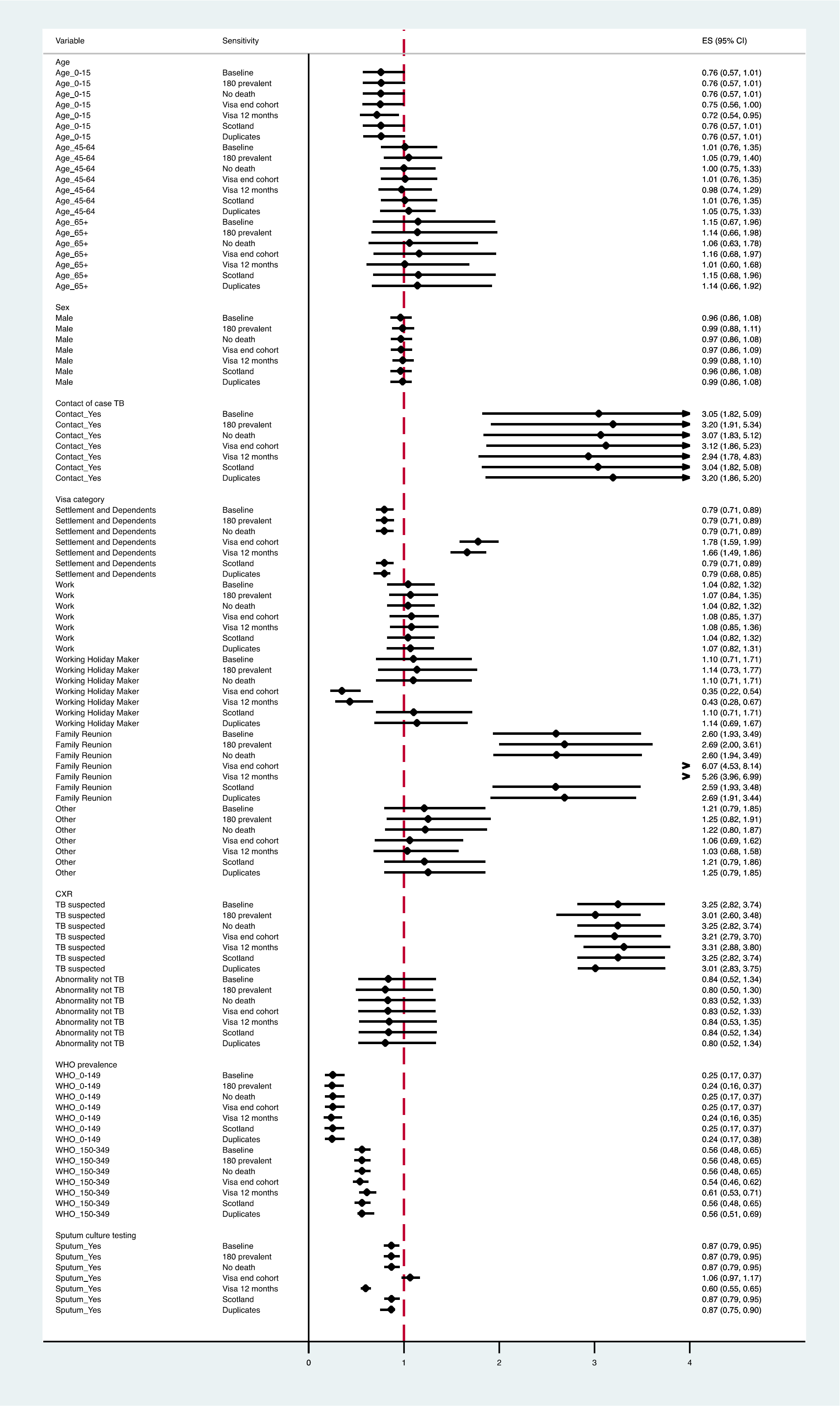

Figure 8. **Sensitivity analysis of multivariable risk factor analysis for all forms of tuberculosis under different model assumptions.**

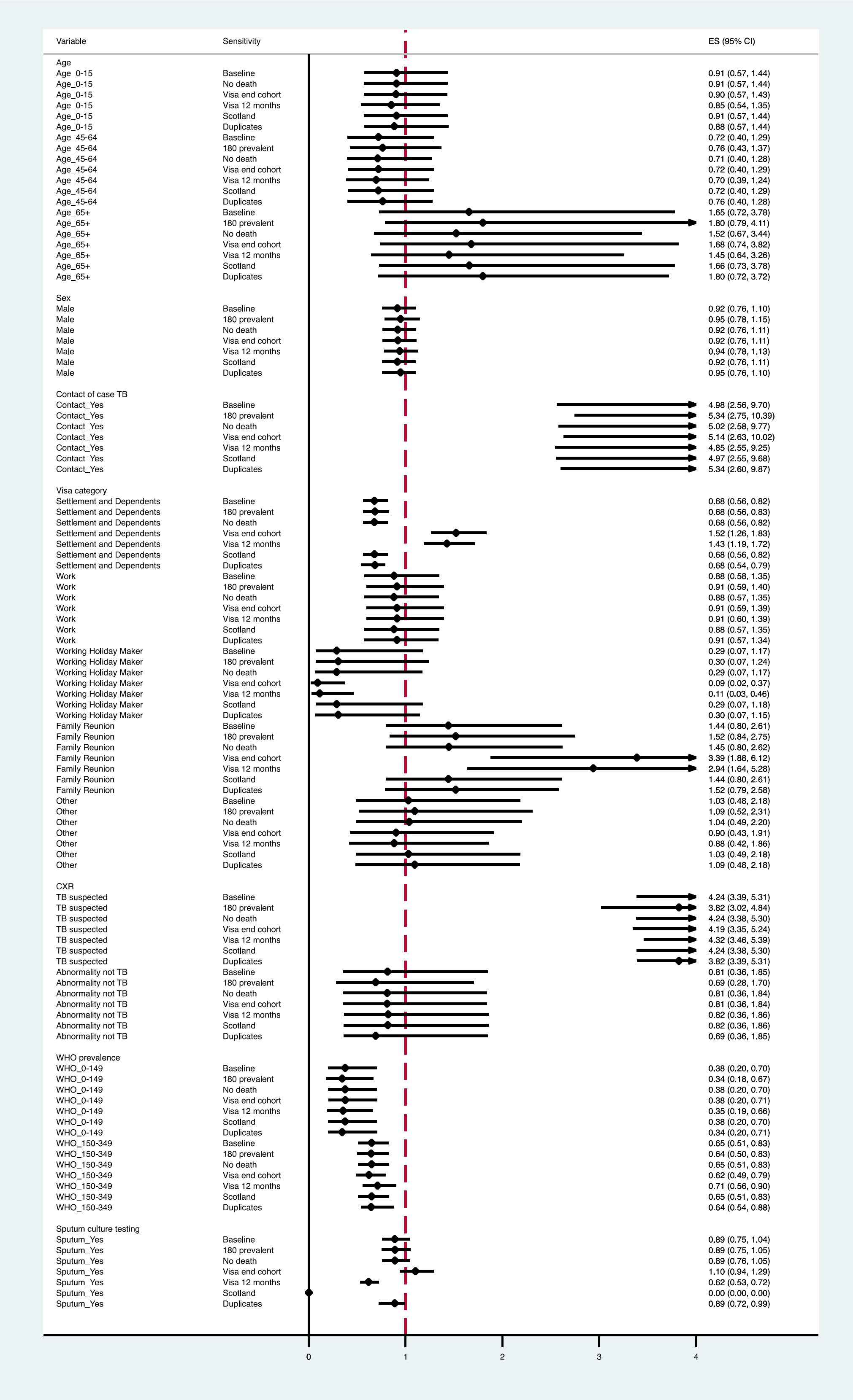

Figure 9. **Sensitivity analysis of multivariable risk factor analysis for bacteriologically confirmed cases of pulmonary tuberculosis under different model assumptions.**

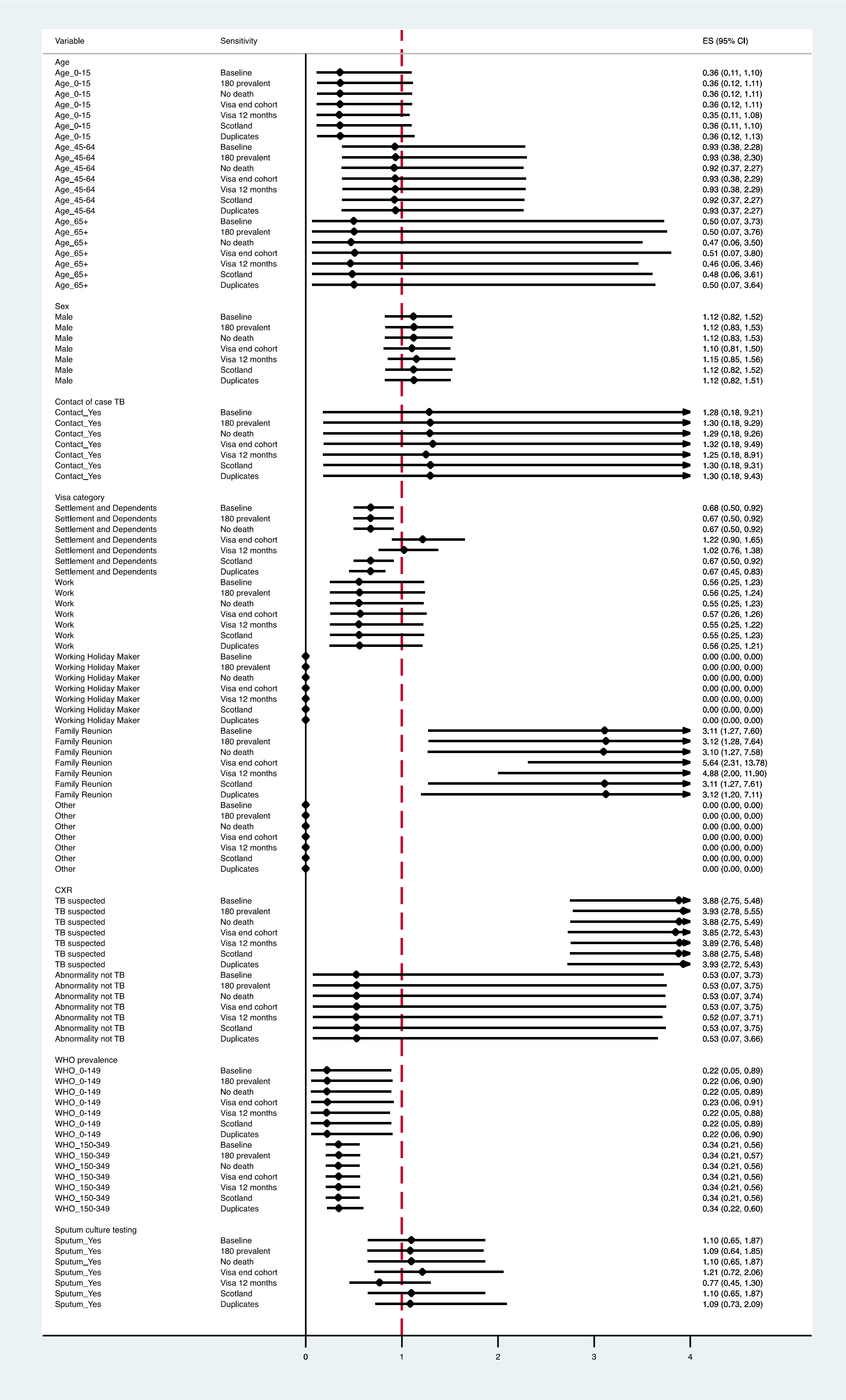

Figure 10. **Sensitivity analysis of multivariable risk factor analysis of incidence rates for assumed reactivation under different model assumptions.**

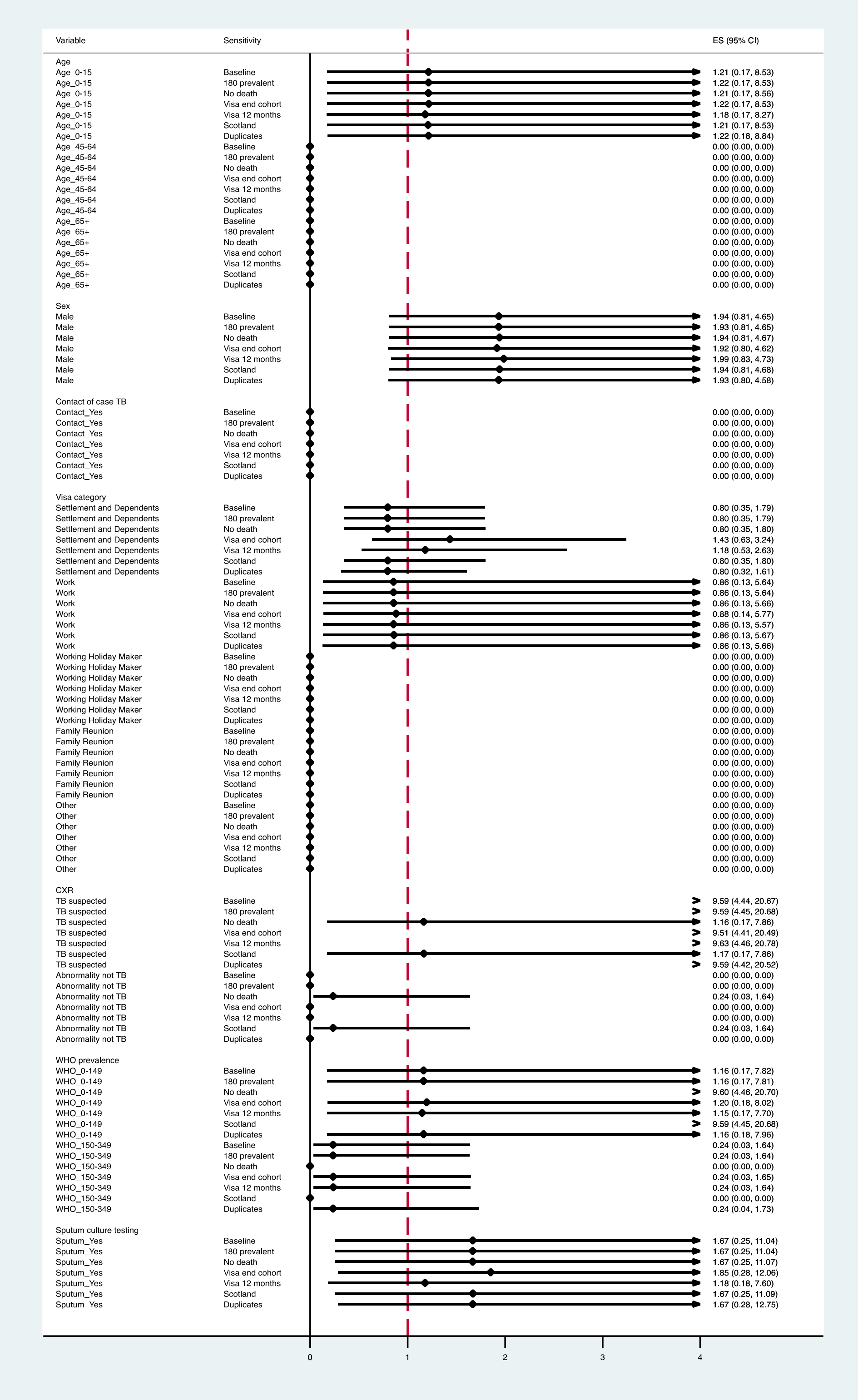

Figure 11. Sensitivity analysis of multivariable risk factor analysis of incidence rates for assumed index cases under different model assumptions.

A final sensitivity analysis was performed to examine the effect of the duration of follow-up available to determine whether cases were clustered or not (Figure 12). As the time window increased from one year of strain typing data (2010) up to four years of strain typing data (2010-2013) the proportion of cases clustered increased from 48.3% (95%CIs: 46.6, 50.0; 3,494 cases included) to 53.5% (95%CIs: 52.8, 54.3; 16,602 cases included). There was no evidence that clustering increased when including four years of data (2010-2013) compared to three years of data (2010-2012) when it was estimated that 53.1% of cases were clustered (95%CIs: 52.3, 54.0; 12,764 cases included).

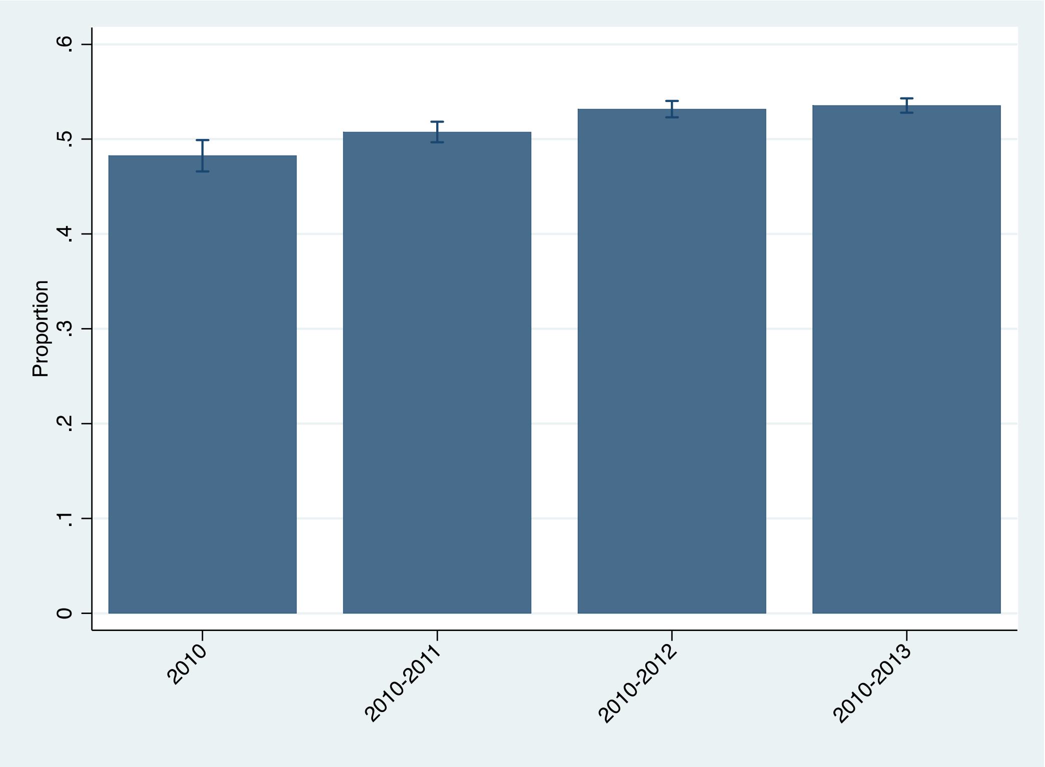

Figure 112. Proportion of all cases clustered by time window.

References

1. Aldridge RW, Yates TA, Zenner D, White PJ, Abubakar I, Hayward AC. “Pre-entry screening for tuberculosis” commentary: authors’ response. Pathog Glob Health. 2015 Jun;109(4):166–7.

2. Ilona C, Natasha H. Introduction To Epidemiology. McGraw-Hill Education (UK); 2011. 202 p.

3. Entry clearance visas by length - GOV.UK [Internet]. [cited 2015 Mar 28]. Available from: https://www.gov.uk/government/publications/entry-clearance-visas-by-length/entry-clearance-visas-by-length

4. Immigration statistics, January to March 2015 - GOV.UK [Internet]. [cited 2016 Feb 15]. Available from: https://www.gov.uk/government/publications/immigration-statistics-january-to-march-2015/immigration-statistics-january-to-march-2015#study-1

5. Immigration statistics, January to March 2015: data tables - Publications - GOV.UK [Internet]. [cited 2016 Feb 15]. Available from: https://www.gov.uk/government/statistics/immigration-statistics-january-to-march-2015-data-tables

6. Cancer Research UK Cancer Survival Group. Tools for Cancer Survival Analysis [Internet]. 2015 [cited 2015 Mar 27]. Available from: http://www.lshtm.ac.uk/eph/ncde/cancersurvival/tools/index.html#

7. ONS. Long Term International Migrants Area of Destination or Origin within the UK, 1991-2013 [Internet]. 2010 [cited 2015 Mar 28]. Available from: http://www.ons.gov.uk/ons/publications/re-reference-tables.html?edition=tcm%3A77-346438
